# Tight regulation of plant immune responses by combining promoter and suicide exon elements

**DOI:** 10.1093/nar/gkv655

**Published:** 2015-07-02

**Authors:** Tania L. Gonzalez, Yan Liang, Bao N. Nguyen, Brian J. Staskawicz, Dominique Loqué, Ming C. Hammond

**Affiliations:** 1 Department of Molecular & Cell Biology, University of California, Berkeley, CA 94720, USA; 2 Joint BioEnergy Institute, 5885 Hollis St, Emeryville, CA 94608, USA; 3 Physical Biosciences Division, Lawrence Berkeley National Laboratory, Berkeley, CA 94720, USA; 4 Department of Integrative Biology, University of California, Berkeley, CA 94720, USA; 5 Department of Plant & Microbial Biology, University of California, Berkeley, CA 94720, USA; 6 Department of Chemistry, University of California, Berkeley, CA 94720, USA

## Abstract

Effector-triggered immunity (ETI) is activated when plant disease resistance (R) proteins recognize the presence of pathogen effector proteins delivered into host cells. The ETI response generally encompasses a defensive ‘hypersensitive response’ (HR) that involves programmed cell death at the site of pathogen recognition. While many R protein and effector protein pairs are known to trigger HR, other components of the ETI signaling pathway remain elusive. Effector genes regulated by inducible promoters cause background HR due to leaky protein expression, preventing the generation of relevant transgenic plant lines. By employing the HyP5SM suicide exon, we have developed a strategy to tightly regulate effector proteins such that HR is chemically inducible and non-leaky. This alternative splicing-based gene regulation system was shown to successfully control Bs2/AvrBs2-dependent and RPP1/ATR1Δ51-dependent HR in *Nicotiana benthamiana* and *Nicotiana tabacum*, respectively. It was also used to generate viable and healthy transgenic *Arabidopsis thaliana* plants that inducibly initiate HR. Beyond enabling studies on the ETI pathway, our regulatory strategy is generally applicable to reduce or eliminate undesired background expression of transgenes.

## INTRODUCTION

Plant disease and health are constant concerns for the agricultural industry and for worldwide food security. As climate change alters the agricultural landscape and creates more favorable conditions for plant pathogens, diseases that have been confined to sub-regions of the world may gain broader geographic footholds (1,2). Understanding how plants successfully combat pathogens is crucial to maintaining healthy agriculture. Plants recognize pathogen associated molecular patterns (PAMPs) such as bacterial flagellin and fungal chitin, and initiate PAMP-triggered immunity (PTI) responses (3,4). However, successful pathogens circumvent this primary innate immune system of plants by delivering effector proteins into plant cells to target and suppress the immune signaling pathway (5). Effector-triggered immunity (ETI) is a secondary plant innate immune pathway that is activated when plants possess disease resistance (R) genes that recognize specific pathogen effector proteins. The ETI response is associated with a hypersensitive response (HR) that involves localized cell death and generation of reactive oxygen species, thereby limiting growth of the pathogen. Although many R protein and effector protein pairs are known to trigger HR and disease resistance, the signaling events that lead to ETI and how much these pathways differ from PTI remains unclear; a lot more is known about how the pathways overlap (4–7).

In order to distinguish specific effector-triggered responses from PAMP-triggered responses, it would be beneficial to generate transgenic plants with an R gene that can inducibly express the corresponding pathogen effector protein to initiate the immune response. In addition, experiments with these types of transgenic plants would enable monitoring of transcriptional and biochemical differences at specific and short time points. However, part of the challenge facing ETI research is the limitation imposed by the HR phenotype. Bacterial, oomycete and fungal pathogens generally produce effector proteins in their own cells and then deliver them into the host plant (4,8–10). Thus, very low thresholds (11,12) of pathogen effector protein are required to trigger HR, which makes generation of transgenic plants for mutant screens and transcriptome analysis difficult (13).

One strategy employed in tomato is to cross resistant plants harboring only the R gene with susceptible plants harboring only the effector gene and then germinating the F1 seeds at elevated temperatures (32–33°C) to suppress HR (13,14). Lowering the temperature then induces HR, but this method is not suitable for all R/effector pairs (13–15), sometimes alters rather than suppresses the HR phenotype (15) and may result in pleiotropic effects such as changes in hormone levels (16). Also, it has only been used for very young plants and cannot generate continuing transgenic lines (13,14).

To our knowledge, the dexamethasone (Dex)-inducible plasmids developed by Aoyama and Chua, pTA7001 and pTA7002 (17), are the major method that has been able to generate continuing transgenic lines expressing both R/effector gene pairs required to induce HR. McNellis *et al.* were able to generate transgenic *Arabidopsis thaliana* plants to study the RPS2/AvrRpt2-dependent ETI pathway by regulating the effector AvrRpt2 with a Dex-inducible promoter (18). The *RPM1* resistance gene has also successfully tolerated inducible regulation of its recognized effectors, including Dex-inducible *avrB* or *avrRpm1*, or estradiol-inducible *avrRpm1* (11,19). *A. thaliana* expressing both the *RPS5* resistance gene and Dex-inducible *avrPphB* is also viable (20). Unfortunately, promoter regulation is not enough to generate transgenic plants for all R/effector pairs. In our experience, healthy and viable *Nicotiana benthamiana* plants expressing Bs2/AvrBs2 or *A. thaliana* plants expressing RPP1/ATR1 have not been able to be generated. Anecdotally, additional examples have been encountered by other researchers, but these negative results are difficult to publish. Beyond applications in immunology research, leaky protein expression from inducible promoters is a common limitation in transgenic plant generation (21).

**Figure 1.**
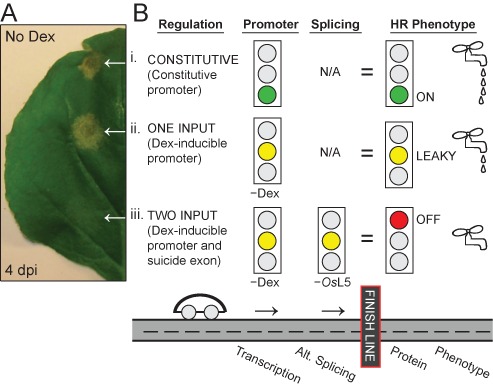
Dual transcription and splicing regulation eliminates leaky hypersensitive response. (**A**) *Nicotiana benthamiana* leaf with three spots transiently co-transformed with *Agrobacterium tumefaciens* to introduce *SuperPromoter*::*Bs2-HA* resistance gene and either (i) constitutive (35S) *avrBs2-HA*, (ii) Dex-inducible (*6xUAS*) *avrBs2-HA* or (iii) Dex-inducible *avrBs2-HyP5SM-HA*. The leaf was not treated with Dex and *OsL5* is not co-expressed. (**B**) Scheme representing how a two input system that combines promoter and suicide exon elements to control transcription and alternative splicing, respectively, results in tighter regulation of phenotype compared to a one input system (promoter alone). See Supplementary Figure S1 for a related scheme showing the case in the presence of inputs.

**Figure 2.**
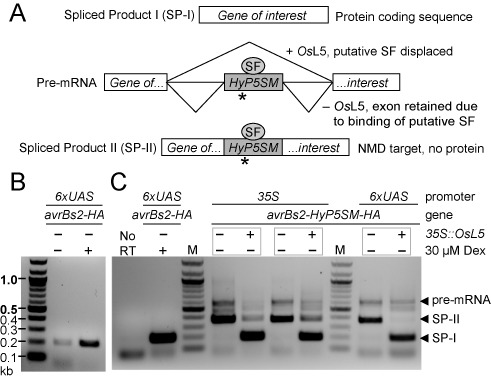
HyP5SM functions in bacterial effector gene *avrBs2-HA*, but does not eliminate leaky transcription. (**A**) Model of HyP5SM splicing regulation by *Os*L5 displacement of an endogenous splice factor (SF) that promotes exon retention by default. A premature termination codon (asterisk) is present in the HyP5SM suicide exon. (**B**) RT-PCR detection of *avrBs2-HA* transcripts from transiently transformed *Nicotiana benthamiana* leaves with and without Dex induction of transcription at 16 hours post-infiltration (hpi), with tissue collected at 2 dpi. (**C**) RT-PCR detection of spliced products for *avrBs2-HyP5SM-HA* with or without *Os*L5 induction of exon skipping, from tissue collected 2 dpi. Results are shown for the construct expressed either using 35S or 6xUAS promoters without Dex induction. After 35 cycles of PCR amplification, the 35S samples appear to be saturated, so levels should not be compared to the 6xUAS samples. No RT is shown as a negative control and *avrBs2-HA* from a different experiment is shown as a positive control for SP-I. Gray boxes indicate samples taken from different infiltrated halves of the same leaf.

Herein, we describe employing a suicide exon in order to tightly regulate the expression of effector proteins, such that the HR is chemically inducible but background HR is fully suppressed (Figure 1, Supplementary Figure S1). Previously, we have shown that insertion of the HyP5SM splicing cassette (Figure 2a) into any gene of interest results in retention of a suicide exon by default, which triggers nonsense-mediated decay of the non-productive spliced product (22,23). Alternatively, co-expression with *Os*L5 protein results in skipping of the suicide exon (22). In this study, we establish that HyP5SM circumvents the problem of leaky transcription from a Dex-inducible promoter by effectively blocking protein expression. Whereas background levels of the effector AvrBs2 from the bacterial spot disease pathogen *Xanthomonas euvesicatoria* (24), previously called *Xanthomonas campestris* pv. *vesicatoria* (25), triggers visible HR in plants harboring the R gene *Bs2* (26), the immune response is suppressed by insertion of the HyP5SM splicing cassette into the *avrBs2* gene. Furthermore, we show that skipping of the suicide exon can be triggered by Dex induction of *Os*L5 expression, which leads to recovery of the HR phenotype by chemical activation of both the promoter and alternative splicing. We demonstrate that this dual regulation strategy is generalizable by showing that similar results are obtained for ATR1, an effector from the downy mildew pathogen *Hyaloperonospora arabidopsidis*, which triggers HR in tobacco plants harboring the R gene *RPP1* (27–29). Finally, we show that stably transformed plants harboring the resistance gene and the dual regulated effector are viable, healthy, can be propagated through multiple generations and can inducibly initiate the HR phenotype.

## MATERIALS AND METHODS

### Oligonucleotides and DNA constructs

Sequences for all synthetic primers used for performing polymerase chain reaction (PCR) and RT-PCR experiments are described in Supplementary Table S1. The HyP5SM splicing cassette sequence was inserted into constructs by overlap extension PCR as discussed in Supplementary Methods (Supplementary Figure SM1) and a previous publication (22). For *avrBs2* constructs, silent mutations to the proximal codons were introduced to conform to the extended splice site consensus and are indicated in Supplementary Table S1. Effector constructs were cloned into the pBinAR vector (Kan^R^) (30) for constitutive expression using the CaMV 35S promoter and into the pTA7001 vector (Kan^R^) (17) for Dex-inducible expression using the 6xUAS promoter. The *avrBs2-HA* gene was cloned from pMD1 *avrBs2-HA* (31) and *ATR1Δ51-FLAG* (Emoy2 allele) was cloned out of pENTR/D-TOPO *ATR1Δ51-FLAG* (29). The Staskawicz lab provided the pTA7001 empty vector (originally from Aoyama and Chua) (17), the pEG202 *35S::ATR1Δ51-FLAG* vector (29) and the R gene vectors, p1776 *Bs2-HA* and pEG301 *pRPP1*::*RPP1–3xHA* (WsB allele) (29). The *Bs2-HA* gene was driven by a constitutive chimeric octopine and manopine synthase promoter (‘SuperPromoter’) (32,33) in the p1776 vector (Kan^R^) (34). The SuperPromoter results in higher expression than the 35S promoter (32). The HyP5SM cassette, pBinAR *OsL5* and control pBinAR *firefly luciferase (LUC)* are from the original HyP5SM paper (22). *OsL5* and *OsL5–6xHis* were amplified from pBinAR *OsL5* and inserted into pTA7001 by restriction digest cloning.

### *Agrobacterium*-mediated transient leaf transformations

*Agrobacterium tumefaciens* strain GV2260 (Rif^R^, Carb^R^) was used for all plasmids in the AvrBs2 experiments except for p1776 *Bs2-HA. A. tumefaciens* strain GV3101 (Rif^R^, Gent^R^) was used for p1776 *Bs2-HA*, the multi-gene pTKan vectors and all plasmids in the ATR1Δ51 experiments. *Nicotiana benthamiana* plants were grown 3 weeks in a greenhouse (Oxford Tract Greenhouse Facility), then transferred to an open growth cart under constant light and ambient temperatures (24–27°C) for ∼1 week before experiments. *Nicotiana tabacum* var Turk plants were grown in a growth chamber under 16/8 h day/night cycles, then transferred to the open growth cart 2 days before experiments.

*Agrobacterium* was grown in LB Miller broth with appropriate antibiotics (all 50 μg/ml) at 28°C. *Agrobacterium* was grown in 5 ml starter cultures overnight, then added to 30 ml selection media and grown for another 20–24 h. All liquid cultures were shaken at 225 rpm. Cells were collected with 10 min 4700 rpm. Cells were washed (10 mM MES, 10 mM MgCl_2_, pH 5.6), then collected again. Cells were resuspended with induction buffer (wash buffer + 150 μM acetosyringone) and incubated for 2–3 h. Cell density was normalized with induction buffer to OD_600_ = 0.5 or 0.75. Normalized Agro solutions were mixed 1:1 or 1:1:1, for a final OD_600_ = 0.25 of each construct. R gene was included in HR experiments and omitted for RT-PCR and western blot experiments to avoid HR. Plant leaves were infiltrated with *Agrobacterium* solutions using a 1 ml needle-less syringe.

### Dexamethasone induction and tissue collection

Dexamethasone (Sigma, CAS 50–02–2) was dissolved in ethanol to make a 30 mM stock solution. *Nicotiana* plants were induced with 30 μM dexamethasone (+0.1% ethanol, in Agro wash buffer) at 16-18 hpi, kept in the dark with restricted air circulation for 2–4 h, then returned to regular growth conditions with constant light. For *N. benthamiana*, injecting with a needle-less syringe or thoroughly spraying the leaves were both effective induction methods in our hands, but spraying was preferred because it caused less tissue damage. For *N. tabacum*, only induction by needle-less syringe was tested. Transgenic *A. thaliana* plants were similarly induced by needle-less syringe with 15 μM dexamethasone or spraying with 30 μM dexamethasone.

At 8–72 h post-dexamethasone induction (hpd), 2–4 leaf discs were collected using the end of a 1 ml pipette tip (∼15–30 mg tissue), snap frozen in liquid nitrogen, and stored at −80°C until ready for processing. RNA or crude proteins were extracted from tissue samples pulverized with stainless steel beads and TissueLyzer II (Retsch) for 25 Hz for 30 s in pre-cooled adapters.

### RT-PCR

RNA was extracted from pulverized tissue using Universal RNA Purification Kit (CHIMERx) with RL buffer. RNA was treated with RQ1 DNase (Promega), then 400–1000 ng of RNA and oligo(dT) primer were used for cDNA synthesis using iScript Select cDNA Synthesis kit (BioRad) or SuperScript III Reverse Transcriptase (Life Technologies). Splicing was assessed using PCR with Taq polymerase (New England Biolabs) and primers designed ∼100 bp outside the splice sites such that the size of amplified SP-I would be approximately 200 bp. Primers are described in Supplementary Table S1.

### Protein extraction, western blots and Ponceau S stains

Total protein was extracted from pulverized tissue using 200–300 μl of Laemmli (35) Buffer (0.24 M Tris–Cl pH 6.8, 6% sodium dodecyl sulphate, 30% glycerol, 16% β-mercaptoethanol, 0.006% bromophenol blue, 10 M urea) as previously described (29). Extract was run on denaturing 10% or 4–12% Bis-Tris NuPAGE gels with MOPS buffer (Life Technologies), then transferred to nitrocellulose membrane using a tank transfer at 300 mA for 1.5 h (for AvrBs2-HA) or 1 h (for ATR1Δ51-FLAG or *Os*L5). Membranes were stained for total protein with Ponceau S (0.5% Ponceau S, 1% acetic acid) and the large subunit of RuBisCo (approx. 55 kDa) is shown below western blots as a loading control. Membranes were blocked overnight with 5% milk in TBST buffer (20 mM Tris–Cl, 0.5 M NaCl, 0.05% Tween-20, pH 7.5). AvrBs2-HA protein was detected using 1:1000 of rat Anti-HA HRP-conjugated antibody (Roche, 3F10). ATR1Δ51-FLAG was detected using 1:2000 of mouse Anti-FLAG M2 HRP-conjugated antibody (Sigma, A8592). Antibodies for *Os*L5–6xHis were made by expressing and purifying exotoxinA-*Os*L5–6xHis, then submitting this protein to a commercial vendor (Josman, LLC) for generation of *Os*L5–6xHis-specific antibodies in rabbits. *Os*L5 protein was detected using rabbit anti-*Os*L5–6xHis 1:40,000 and goat anti-rabbit HRP-conjugated 1:5000 (BioRad, 170–6515).

### Generation of transgenic *A. thaliana* plants

To facilitate the introduction of multiple genes into *A. thaliana*, pTKan binary vectors were designed (Supplementary Table S2, Figure SM2) and constructed as described in Supplementary Methods. *A. thaliana* Col-0 ecotype plants were grown in a growth chamber on long day conditions (16/8 h day/night cycles) and transformed using the *Agrobacterium*-mediated floral dip method (36). Briefly, *Agrobacterium tumefaciens* strain GV3101 containing the binary pTKan vectors were cultured in LB Lennox (low salt) media for 16–24 h at 28°C, 225 rpm, with rifampicin (50 μg/ml), gentamycin (50 μg/ml) and kanamycin (50 μg/ml) selection. Cells were pelleted at 4700 rpm for 15 min and resuspended in floral dip buffer (5% sucrose, 10 mM MgCl_2_, 10 mM MES buffer pH 5.6, 0.018% Silwet L-77). The flowering stems of young *A. thaliana* plants were dipped in the *Agrobacterium* solution for 30–60 s. Plants were left to recover in the dark for 16–24 h, grown on long days to maturity, then dried and the seeds were collected by sifting.

To select for transgenic plants, the seeds of floral-dipped plants were ethanol-sterilized and plated on phytoagar media in a sterile biosafety cabinet. Phytoagar media was prepared as follows: 2.16 g/l MS, 20 g/l sucrose, 0.35 g/l MES, water added, pH to 5.7–5.8 and then 7.5 g/l phytoagar added. Media was autoclaved for 30 min, filter-sterilized kanamycin (50 μg/ml) and cefotaxime (200 μg/ml) were added once media had cooled, then plates were poured and allowed to set inside the sterile cabinet. Phytoagar plates with seeds were sealed with breathable microfilm, wrapped in foil and stored at 4°C for 2–4 days. Plates were unwrapped and transferred to a growth chamber for germination under long days. Transgenic plants are viable with kanamycin treatment and were transferred to soil at 10 days, then rosette leaves were scanned for expression of the DsRed2 reporter gene using a Typhoon laser image scanner (ex/em: 532/580 nm). DsRed2 fluorescence was used to estimate expression of the other transgenes, thus reducing the initial labor required to analyze and compare independent lines. Lines expressing DsRed2 fluorescence were carried forward. Subsequently, expression of other transgenes was verified by RT-PCR.

*Arabidopsis thaliana* 3860 ecotype plants homozygous for *pRPP1::RPP1-WsB-3xHA::tRPP1* (Basta resistant, kanamycin sensitive) were acquired from the Staskawicz group (37), and transformed with pTKan C59 (Supplementary Figure SM2) by floral dip. The resulting transgenic plants are Basta and Kan resistant and carry copies of the R gene *RPP1*, the effector gene *ATRΔ51-HyP5SM-FLAG* (Emoy2 allele, inducible), *OsL5–6xHis* (inducible), the chimeric transcription factor GAL4 DNA binding domain-VP16 activating domain-Glucocorticoid receptor regulatory domain (GVG) and *DsRed2*. As a control, homozygous Col-0, which does not recognize the Emoy2 allele of *ATR1*, was transformed with the pTKan C59 vector. Data shown for plants carry both effector and R genes are for T2 lines (either hemizygous or homozygous). Control plants carrying just the *ATR1* effector gene or the *RPP1* R gene are homozygous lines.

## RESULTS

### HyP5SM is functional in bacterial effector AvrBs2 and eliminates detectable leaky protein expression that leads to background hypersensitive response

**Figure 3.**
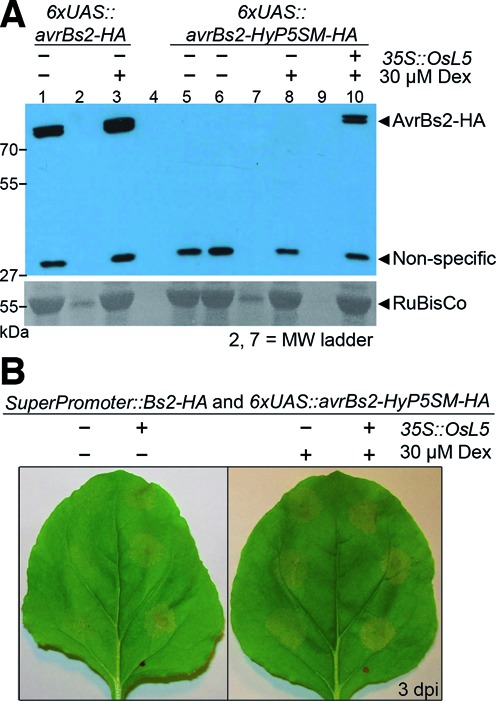
The two-input regulatory system overcomes both leaky transcription and exon skipping to tightly control protein expression and HR phenotype. (**A**) Anti-HA western blot to detect protein expression from *avrBs2-HA* or *avrBs2-HyP5SM-HA* constructs induced or mock induced at 18 hpi, with tissue collected at 26 hpi. Shown at the bottom is Ponceau S staining of the nitrocellulose membrane to visualize the large subunit of RuBisCo as a loading control. (**B**) Induction of the HR phenotype from the effector construct *avrBs2-HyP5SM-HA* requires co-transformation with *Bs2-HA* and either co-transformation with *OsL5*, treatment with 30 μM Dex or both. Neither chlorosis nor HR is visible in the absence of both inputs. Lanes 4 and 9 are empty.

AvrBs2 is a Type III secreted effector protein highly conserved and required for virulence in strains of the bacterial spot disease pathogen *X. euvesicatoria* (24). The *Bs2* disease resistance gene from *Capsicum chacoense* (pepper) is functional in *Solanum lycopersicum* (tomato) and increases the yield of marketable tomatoes by 2.5-fold in field tests (26,38). Unfortunately for studying this effector-R gene pair, we have found that leaky transcription from the Dex-inducible 6xUAS promoter (17) results in background expression of AvrBs2 protein, which activates HR in *N. benthamiana* plants co-transformed with Bs2 (Figure 1a). Accordingly, even without Dex treatment, *avrBs2* transcripts are detectable by RT-PCR (Figure 2b) and HA-tagged AvrBs2 protein is observed by western blot (Figure 3a). Thus, HR has prevented the generation of stable transgenic plants that inducibly express AvrBs2 in the presence of Bs2 (D. Dahlbeck and B. J. Staskawicz, unpublished results).

In order to address this problem, we inserted the HyP5SM splicing cassette into the *avrBs2* coding sequence (Supplementary Figure S2). It was previously shown that the splicing cassette minimally requires insertion after an AG dinucleotide which forms part of the extended 5′ splice site sequence (22). Cassette insertion between codons encoding Glu and Val (E/V; GAG/GTA) as well as Glu and Pro (E/P; GAG/CCA) had been well tolerated in the context of *Enhanced Green Fluorescent Protein (EGFP)*, which reveals that the extended 3′ splice site could deviate substantially from the consensus sequence (22). However, in the context of the *avrBs2* coding sequence, splicing cassette function appears more sensitive to the extended 3′ splice site context. Specifically, placing the splicing cassette between codons encoding E308 and V309 resulted in maintenance of splicing fidelity and regulation (Figure 2c, Supplementary Figure S2), whereas mis-splicing was observed for insertion between codons encoding E123 and P124 (Supplementary Figure S3). Thus, we continued with the E/V insertion site for *avrBs2-HyP5SM-HA* and all text references relate to this construct unless otherwise stated.

RT-PCR analysis of *avrBs2-HyP5SM-HA* show that in the absence of the specific splicing factor *Os*L5, which is ribosomal protein L5 from the monocot *Oryza sativa* (rice) that binds the P5SM RNA element (22), the suicide exon is retained in the spliced product (SP-II) (Figure 2c). As expected, endogenous L5 protein does not result in observable background activation of the splicing cassette, as the HyP5SM cassette was engineered to be unresponsive to L5 from dicots such as *N. benthamiana* and *A. thaliana* (22). In contrast, constitutive co-expression of *Os*L5 promotes skipping of the suicide exon and leads to a shorter spliced product (SP-I, Figure 2). These results were confirmed by sequence analysis of the spliced products and are observed regardless of whether the gene construct is expressed via a constitutive (35S, a cauliflower mosaic virus promoter) or Dex-inducible (6xUAS) promoter. We did not observe any of the splicing isoforms previously associated with aberrant splicing of the cassette in weak sequence contexts (22), however this analysis does not rigorously exclude all possible mis-splicing events.

Notably, the HyP5SM splicing cassette does not reduce leaky transcription from the Dex-inducible promoter, as transcripts are clearly observed by RT-PCR in the absence of Dex (Figure 2c). However, HyP5SM adds a second layer of regulation such that induction of alternative splicing is required for protein expression. Thus, we have nominally constructed a two-input regulatory system in which the chemical inducer Dex and the splicing factor *Os*L5 are required for full activation of gene expression (Figure 1b, Supplementary Figure S1). However, the observation that HR can be triggered with only one input (Figure 3b, Supplementary Figure S4) suggests that both transcription and exon skipping are leaky to some extent. The sensitivity of HR signaling is such that cell death is visible even when no protein is detected by a standard western blot (Figure 3a, lane 8). Additional experiments confirm that some exon skipping occurs upon Dex induction even without *Os*L5, presumably because the transcription is activated in excess of the levels of the endogenous splice factor that promotes exon retention (Supplementary Figure S5). More importantly, the absence of both Dex and *Os*L5 eliminates HR (Figure 3b). Only mild chlorosis is observed on that leaf half, similar to results obtained for leaf infiltration with *Agrobacterium* harboring no AvrBs2 effector (Supplementary Figure S6).

### Tight regulation of the HR phenotype by dual chemical induction of transcription and exon skipping

Although omission of the splicing factor effectively suppresses protein expression from the HyP5SM-containing *avrBs2* construct, we considered that transgenic plant experiments would instead require an inducible copy of *Os*L5. However, as the inducible promoter employed would likely be leaky, it was an open question whether background expression of *Os*L5 would trigger sufficient AvrBs2 protein expression to cause HR. To test this, we performed the subsequent experiments with *Os*L5 under the control of 6xUAS. In this case, the two-input regulatory system is constructed such that the same chemical inducer Dex serves as both the input for activation of transcription and exon skipping (Supplementary Figure S1d).

**Figure 4.**
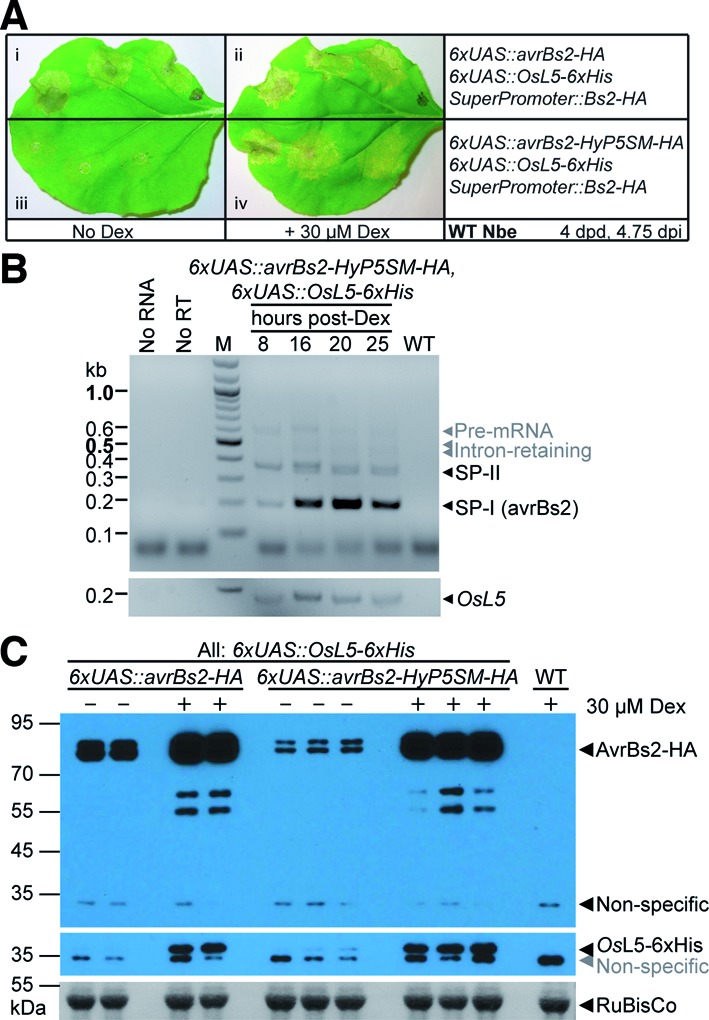
Comparison of single and dual regulation by chemical induction. (**A**) Representative wild-type *Nicotiana benthamiana* leaves were spot infiltrated to compare the extent of HR phenotype from (i, ii) pTA7001 *avrBs2-HA* (only regulated by the Dex-inducible promoter) or (iii, iv) pTA7001 *avrBs2-HyP5SM-HA* (also includes splicing regulation), with or without 30 μM Dex treatment. Leaf spots were also co-infiltrated with pTA7001 *OsL5–6xHis* and p1776 *Bs2-HA*. (**B**) Top: RT-PCR detection of *avrBs2-HyP5SM-HA* transcripts from transiently transformed *N. benthamiana* leaves at different time points post induction with Dex. Controls shown are no RNA or no RT enzyme in cDNA synthesis reaction and RT-PCR from wild-type tissue. Bottom: RT-PCR detection of *OsL5* transcripts. (**C**) Top: Anti-HA western blot to detect protein expression from *avrBs2-HA* or *avrBs2-HyP5SM-HA* constructs induced with Dex or mock induced. Samples are from biological replicates. Leaf tissue were also co-infiltrated with pTA7001 *OsL5–6xHis*, except for the wild-type control. Middle: Anti-*Os*L5 western blot. Bottom: Ponceau S stain of the large subunit of RuBisCo as loading control.

Promisingly, background expression of *Os*L5 in the absence of Dex does not trigger HR when the two-input *avrBs2-HyP5SM-HA* construct is employed, in contrast to the *avrBs2-HA* construct, which does not contain the splicing cassette (Figure 4a). Chemical induction of *avrBs2-HyP5SM-HA* and *OsL5* still produces the robust HR phenotype, although the onset of visible HR is delayed (<16 h) relative to the *avrBs2-HA* construct. Presumably this delay is due to leaky build-up of effector protein in the latter case, before the *N. benthamiana* leaves are Dex-induced (typically 16–18 hpi). Consistent with this interpretation, a time course analysis shows no detectable AvrBs2 protein expression until 16 h after Dex induction for the two-input system, whereas protein expression was detected as soon as 4 h after Dex induction for the one-input system (Supplementary Figure S7a and b). Induction of high levels of AvrBs2 protein occurs at the same time as expression of *Os*L5 protein in the two-input system (Supplementary Figure S7c).

Correspondingly, RT-PCR and western blot analyses support that chemical induction of *Os*L5 promotes exon skipping to make SP-I the major splice product, which leads to high AvrBs2 expression (Figure 4b and c). Interestingly, in the absence of Dex induction, it appears that leaky expression of *Os*L5 can generate a small amount of AvrBs2 protein from the HyP5SM-containing construct, but the signal is much lower than observed for leaky expression of the *avrBs2-HA* construct (Figure 4c). We showed that this effect is probably due to leaky expression of *Os*L5, because no AvrBs2 protein is observed when co-expressed with empty pTA7001 vector, which carries another copy of GVG transcription factor (Supplementary Figure S7d). Nevertheless, the results suggested that this combined promoter- and splicing-based regulation will be sufficiently tight to generate viable transgenic plants to study immune responses.

### HyP5SM is also functional in oomycete effector ATR1Δ51

Pathogen effectors are recognized by different classes of resistance proteins. The most characterized classes are CC-NB-LRR and TIR-NB-LRR, with a conserved nucleotide binding (NB) site and a leucine rich repeat (LRR) domain for auto-inhibition (39) and effector specificity (40). The N-terminal domain is known to be an important determinant for the downstream signaling pathway, with NDR1 being required for CC-NB-LRR protein signaling and the EDS1/PAD4/SAG1 complex being required for TIR-NB-LRR protein signaling (40–43).

AvrBs2 is recognized by Bs2, which has an uncharacterized N-terminal domain, but is homologous to the *Rx* gene (CC-NB-LRR) from potato (26,44–45). On the other hand, the ATR1 effector from the oomycete *H. arabidopsidis*, agent of downy mildew in *A. thaliana*, is recognized by the *A. thaliana* RPP1 resistance protein, which is in the TIR-NB-LRR class. For efficient expression in plants, we utilized a construct, ATR1Δ51, that has been truncated to remove the N-terminal eukaryotic secretion sequence and predicted translocation region (29). Co-expression of ATR1Δ51 and RPP1 produces weak HR in *N. benthamiana* and strong HR in *N. tabacum* (Supplementary Figure S6), so we utilized the latter for our HR experiments.

**Figure 5.**
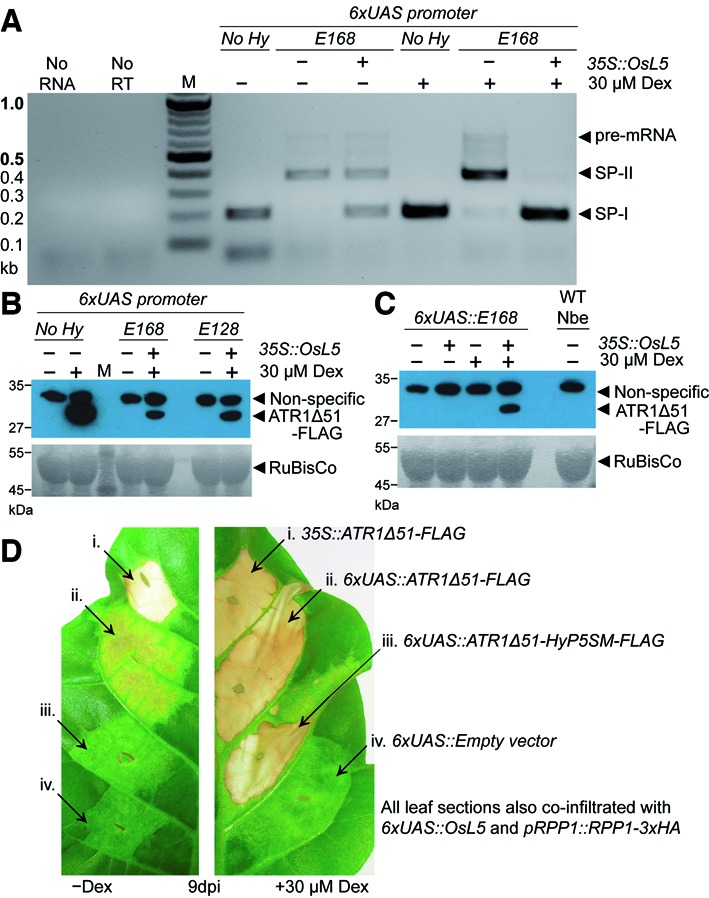
HyP5SM tightly regulates the hypersensitive response induced by the oomycete effector gene *ATR1Δ51-FLAG* in *Nicotiana tabacum*. (**A**) RT-PCR detection of transcripts from *ATR1Δ51-FLAG* (‘No Hy’) or *ATR1Δ51-HyP5SM-FLAG* (‘E168’) transiently transformed in *N. benthamiana* leaves. The constructs were expressed under the control of 6xUAS, with and without Dex induction, and with and without OsL5 co-expression. (**B** and **C**) Anti-FLAG western blot using *N. benthamiana* tissue from the same experiment in (a). The RuBisCo large subunit visualized by Ponceau S staining of the nitrocellulose membrane serves as a loading control. (**D**) Comparison of the HR phenotype in *N. tabacum* for constitutive (i), Dex-inducible promoter (ii) and Dex-inducible promoter and splicing factor (iii) regulated *ATR1Δ51-FLAG*. Empty vector (iv) serves as a negative control. Left panel shows the extent of leaky HR without Dex induction and right panel shows induction of HR with 30 μM Dex.

In order to demonstrate that the HyP5SM splicing cassette can be applied to regulate a different effector protein in both *Nicotiana* species, we inserted the HyP5SM splicing cassette into the *ATR1Δ51* coding sequence. We made two versions that placed the splicing cassette between Glu and Ala codons (E/A sites) after codons encoding E128 or E168 (Supplementary Figure S2). Both constructs maintained splicing fidelity and regulation (Figure 5a, Supplementary Figure S8). We continued with the E168/A169 insertion site for *ATR1Δ51-HyP5SM-FLAG* and all text references relate to this construct unless otherwise stated. Furthermore, regulation of protein expression by Dex induction and co-expression of *Os*L5 was observed by western blot against anti-FLAG (Figure 5b and c). In this case, no protein was detectable in the absence of Dex, showing that the one-input system is less leaky in this case than for AvrBs2 in *N. benthamiana*.

However, background HR in the absence of Dex treatment of *N. tabacum* is still observed for the one-input system upon co-expression of RPP1 and inducible ATR1Δ51 (Figure 5d). Onset of visible HR due to leaky expression is delayed (6–9 dpi) relative to constitutive or induced expression of ATR1Δ51 (2.5–4 dpi). As expected, the dual regulatory system using *6xUAS::ATR1Δ51-HyP5SM-FLAG* and *6xUAS::OsL5* eliminates the problem with background HR. Only mild chlorosis similar to that observed for empty vector, which expresses the GVG transcription factor, is observed up to 13 dpi (Figure 5d, Supplementary Figure S8). Furthermore, induction of HR with 30 μM Dex occurred on a similar timescale and strength for constitutive, one-input and two-input systems (Supplementary Figure S8).

Taken together, these data show that our system can be used to tightly regulate both highly leaky and less leaky effector proteins. Importantly, background ATR1Δ51 levels still exceed the threshold for triggering HR, which appears to be below the detection limit for western blots. In addition, we show that HyP5SM functions robustly in *N. tabacum*.

### HyP5SM enables generation of stable *Arabidopsis* plant lines that inducibly activate the hypersensitive response

In order to demonstrate that our system enables generation of viable transgenic plants, we constructed a multi-gene pTKan vector containing Dex-inducible *OsL5*, Dex-inducible *ATR1Δ51-HyP5SM-FLAG*, 35S::*GVG* (the Dex-responsive transcription factor from pTA7001) and pNOS::*DsRed2* reporter gene in the T-DNA insertion cassette (C59, Supplementary Figure SM2). Transient transformation experiments with the C59 pTKan vector in *N. tabacum* showed that it can promoted HR after Dex induction (Supplementary Figure S9a), although the phenotype was weaker than the HR seen from co-infiltrating pTA7001 *ATR1Δ51-HyP5SM-FLAG*, pTA7001 *OsL5* and pEG301 *RPP1–3xHA* vectors (Figure 5d). The multi-gene pTKan vector contains only one copy of the GVG transcription factor, whereas previous experiments included multiple copies of GVG due to inclusion of separate pTA7001 vectors. Thus, the weaker HR phenotype may be due to titration of the GVG transcription factor or insufficient induction of *Os*L5 protein. Moreover, due to the large T-DNA size of the C59 pTKan vector, full-length T-DNA transfer efficiency might be lower than for the three co-infiltrated vectors. We tested these hypotheses by co-infiltrating pTKan C59 with either pTA7001 empty vector or pTA7001 *OsL5* since GVG transcription factor and *Os*L5 are located in the beginning and middle of the T-DNA of C59 respectively. Adding extra copies of either GVG transcription factor or *OsL5* promoted stronger HR, suggesting that they are limiting factors (Supplementary Figure S9b).

**Figure 6.**
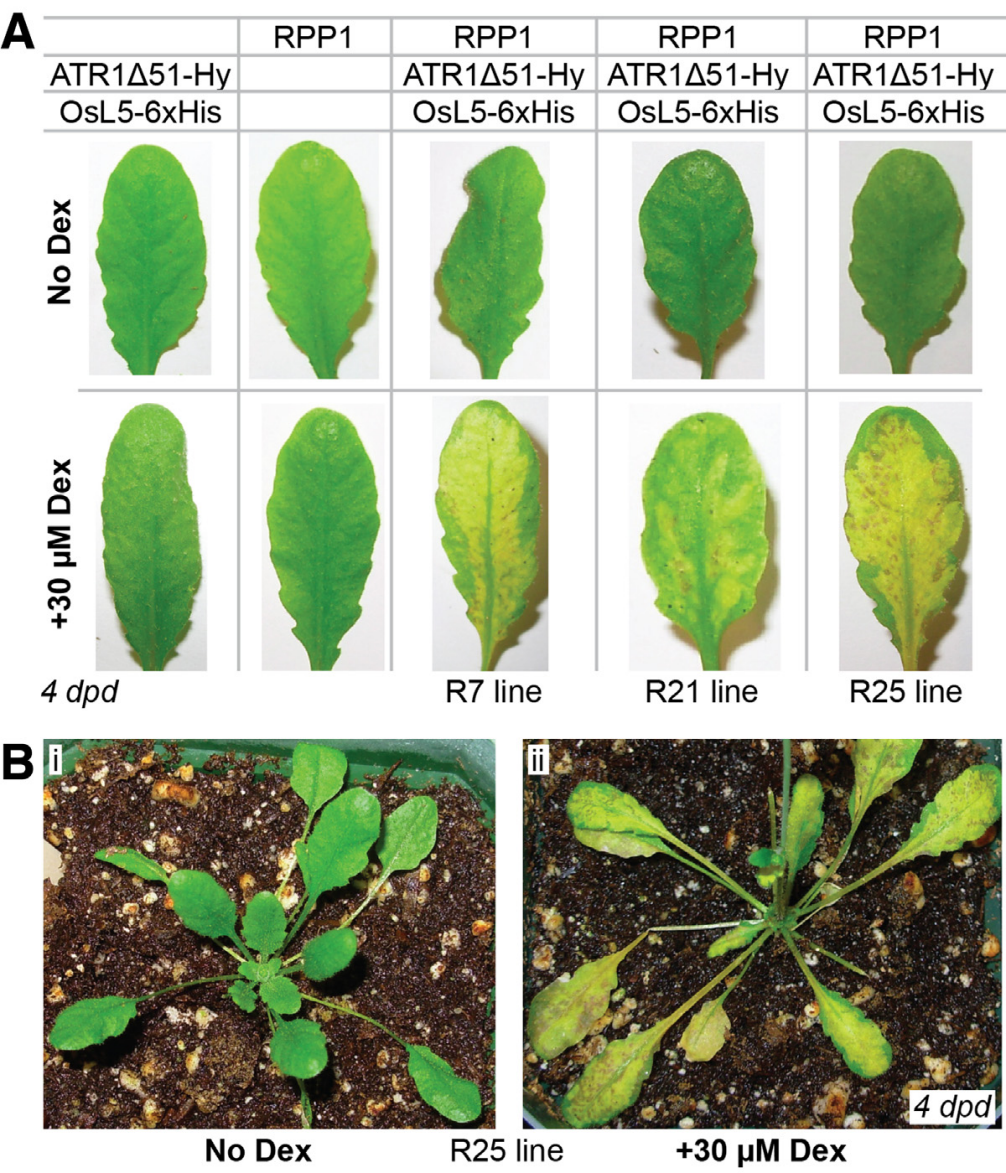
Transgenic *Arabidopsis thaliana* plant lines that contain both the resista­­nce gene and the dual regulated effector gene are viable and the hypersensitive response is chemically inducible. (**A**) Representative leaves from plant lines carrying the effector (ATR1Δ51-HyP5SM-FLAG) and *Os*L5-6xHis, the R gene (RPP1-3xHA), or all three genes, without and with Dex induction. (**B**) Rosettes of line R25 plants without and with Dex induction.

To transform plants with C59, we performed floral dips of *A. thaliana* line 3860 containing *RPP1–3xHA* (WsB allele) acquired from B. J. Staskawicz (37) and of *A. thaliana* Col-0 ecotype, which does not respond to the Emoy2 allele of *ATR1* because it does not harbor the *RPP1* WsB allele. Transgenic seedlings were grown on selection plates and transplanted to soil after 10 days. Plants looked healthy and displayed no obvious growth defects compared to wild type (Figure 6, Supplementary Figure S10). Furthermore, Dex induction of three independent second-generation lines resulted in an RPP1/ATR1-dependent HR characterized by initial chlorosis, browning, tissue collapse and eventual drying of induced leaves (Figure 6, Supplementary Figure S10). RT-PCR analysis confirmed that *Os*L5 and SP-I, the translation-competent spliced product of ATR1, both accumulate after Dex treatment, although the weak induction levels again suggests that some factors are limiting (Supplementary Figure S10c). Injection of individual leaves with 15 μM Dex or spraying the whole plant with 30 μM Dex both resulted in HR (Figure 6, Supplementary Figure S10).

Similarly, we were able to transform *A. thaliana* Col-0 with C93, a multi-gene pTKan vector which contains Dex-inducible *Os*L5, Dex-inducible *AvrBs2-HyP5SM-HA, 35S::GVG*, 35S::*Bs2–3xFLAG* and pNOS::*DsRed2* (Supplementary Figure SM2). These plants were viable and healthy as well. Dex induction resulted in the expected accumulation of transcripts as observed by RT-PCR and a Bs2/AvrBs2-dependent phenotype that was distinct from HR, observed as gradual darkening of the leaves to a purple color (Supplementary Figure S11). The *X. euvesicatoria* pathogen (source of *avrBs2*) does not infect *A. thaliana* and the *Bs2* gene is not known to function in *A. thaliana*, so we are unclear if this is a true or biologically relevant immune response. Nevertheless, these results together demonstrate that the dual regulation system works well in stable transgenic lines.

## DISCUSSION

To our knowledge, this study is the first time that regulation of transcription and exon skipping has been combined to make an artificial two-input regulatory system for use in plants. We show that the dual system is more tightly regulated (i.e. has lower background) than the promoter-only system, as evidenced by suppression of leaky HR (Figure 1a), an immune response sensitive to lower levels of protein expression than a western blot. Importantly, our study demonstrates the general principle that layering regulatory elements reduces the leakiness of gene expression, even when each regulatory element remains leaky. Background transcription from the 6xUAS promoter presumably occurs because some GVG transcription factor enters the nucleus even without binding dexamethasone (Dex). Background skipping of the HyP5SM suicide exon could result from limiting levels of the exon-defining splice factor that is proposed to bind HyP5SM RNA (22) and/or from leaky expression of Dex-inducible *Os*L5. The dual regulatory system reduces leaky gene expression by requiring both transcription and exon skipping for protein production (Figure 1b).

By the same principle, maximum gene expression becomes dependent on both regulatory elements, which has two effects. First, since full activation of exon skipping is dependent on the *Os*L5 protein, which in this case is also expressed using a Dex-inducible promoter, there is a delay in full induction of protein expression (Supplementary Figure S7). In part, this effect is due to the leaky construct having a head-start in protein accumulation relative to the non-leaky construct. Leaves are typically treated with Dex 18 h after infiltration and the observed delay is *<*16 h (Supplementary Figures S4 and S7), consistent with this conclusion. In addition, a threshold of *Os*L5 protein levels in the nucleus must be reached before splicing switches to the exon-skipped product as the major splice product, which likely reflects the equilibrium constants for competitive binding to HyP5SM in the cell. Since alternative splicing requires *Os*L5 binding to each precursor mRNA and the levels of *Os*L5 are probably limiting when activation of *avrBs2* transcription begins, this step may limit or slow build-up of AvrBs2 protein levels. This point is illustrated by comparing western blots when *Os*L5 is driven by the constitutive 35S promoter versus the Dex-inducible 6xUAS promoter. When co-expressed with *35S::OsL5*, AvrBs2 protein levels appear comparable 8 h after Dex induction with or without HyP5SM regulation (Figure 3a), whereas AvrBs2 protein from the two-input system is not yet seen at that time when using *6xUAS::OsL5* (Supplementary Figure S7). Possible solutions to this issue include performing Dex induction twice with optimized timing, or a using different inducible promoter to control *Os*L5 while the gene of interest is still under the Dex inducible system.

Although protein accumulation is more gradual with HyP5SM, chemical induction of the HR phenotype appears to be only slightly delayed (12 h) for Bs2/AvrBs2-dependent HR (Supplementary Figure S4) and not noticeably delayed for RPP1/ATR1Δ51-dependent HR (Supplementary Figure S8). These results suggest that protein accumulation quickly surpasses the threshold required to induce HR. Indeed, we have shown that without *Os*L5 protein, Dex induction still promotes sufficient protein expression from *avrBs2-HyP5SM* to trigger HR (Figure 3b, Supplementary Figure S4 and S7). The option to omit *Os*L5 simplifies the HyP5SM regulation system; researchers may clone the HyP5SM cassette into their gene of interest and utilize pre-existing plasmids with desired promoters, without needing to clone in an additional gene or co-infiltrate with an additional plasmid. However, we recommend that the stability and expression level of the protein of interest be considered before omitting *Os*L5. For example, we found that AvrBs2 protein accumulated to high levels and was easily visualized on a western blot, whereas ATR1Δ51 protein levels were consistently lower. Because of this, stable plant lines were generated using *Os*L5 with the RPP1/ATR1Δ51 system.

Impressively, our two-input system enables the generation of viable, healthy transgenic plants harboring both effector and R genes. We have made independent stable transgenic *A. thaliana* lines carrying dual regulated *ATR1Δ51-HyP5SM* in the presence of *RPP1* and Dex-inducible *OsL5*. We have shown that the HR phenotype is suppressed by default, yet can be chemically induced (Figure 6, Supplementary Figure S10). These transgenic plants will allow us to study *RPP1* disease resistance under different environmental conditions, at different stages of plant development, in different tissues and at short time scales. The broad applicability of the HyP5SM cassette to any gene means that transgenic plant research will no longer be limited to studying the signaling pathways of R genes that can tolerate leaky effector protein expression.

Because omission or inclusion of *Os*L5 affects maximum proteins levels of the HyP5SM-regulated gene (Supplementary Figure S7), HyP5SM regulation is tunable to specific needs. For plant biology studies, it is often desirable to match endogenous levels of gene expression. Chemically inducible promoters can drastically overshoot native expression levels and are difficult to tune. Overexpression can lead to artifacts like loss of biologically-relevant regulation (20). For example, overexpression of Dex-inducible *Pseudomonas syringae* AvrPphB effector protein in transgenic *A. thaliana* resulted in acylation-independent HR in an otherwise acylation-dependent HR pathway (20). In contrast, the dual regulation system leads to moderate induction and even levels of protein expression that lasts at least 72 h after Dex treatment (Supplementary Figure S7). In the future, we are interested in applying this dual regulation to achieve inducible, native levels of transgene expression in plants.

Another key advantage of our system is that it enables two independent promoters to be ‘stacked’ in parallel to regulate a single gene or possibly multiple genes. While here we showed the stacking of two identical promoters, this need not be the case. For example, plant biotechnology applications may involve targeting proteins to specific tissues or cellular compartments, or limiting protein expression to certain developmental stages. This may be achieved by expressing the HyP5SM-regulated gene of interest with a tissue- or stage-specific promoter along with an inducible *Os*L5 gene. Another successful approach to promoter stacking has been described, in which tissue-specific and chemically inducible expression of a gene of interest was effected by stacking two transcription factors/activators. Ethanol induction of GUS expression (*alcA::GUS*) is effected by the AlcR transcription factor under the control of a UAS promoter (*UAS::alcR*), which in turn is recognized by the mGAL4-VP16 transactivator, whose expression was localized to the endosperm in an *Arabidopsis* line from the Haseloff enhancer trap library (46). However, this latter approach is limited in scope to combining chemical induction and tissue specificity, whereas our strategy is generally applicable to combining any type of promoters. In fact, a third layer of regulation may be achieved using our system by changing the promoter driving GVG from the constitutive 35S promoter to make a similar tissue-specific Dex-inducible system.

In conclusion, we have established that the strategy of combining promoter and suicide exon elements leads to tight regulation of pathogen effector proteins and prevents background triggering of even very sensitive phenotypes such as HR. We also showed for the first time that the HyP5SM splicing cassette functions in *N. tabacum* as well as *N. benthamiana*, and we expect that it will function in other dicot plants as well. A similar suicide exon may be engineered for monocot plants in a similar manner as has been described for the current HyP5SM (22) used here. Notably, unlike other inducible expression systems for plants, both the HyP5SM and *Os*L5 sequences are entirely derived from plants (22). If combined with native plant promoters, a fully plant-derived dual regulation system may be constructed. Finally, the dual regulation system we describe is generally applicable to genes that initiate severe or undesired phenotypes upon leaky expression and provides a straightforward and promising way to generate many previously unattainable transgenic plants.

## Supplementary Material

SUPPLEMENTARY DATA
