# Supplementary material for: Tight regulation of plant immune responses by combining promoter and suicide exon elements
Source: Nucleic Acids Res. 2015 Jul 2;43(14):7152–61. doi: 10.1093/nar/gkv655 (PMC4538838; doi:10.1093/nar/gkv655)
Supplement: SUPPLEMENTARY DATA [file supp_43_14_7152__index.html]

Tight regulation of plant immune responses by combining promoter and suicide exon elements — Tight regulation of plant immune responses by combining promoter and suicide exon elements — SUPPLEMENTARY DATA 

# Tight regulation of plant immune responses by combining promoter and suicide exon elements

## SUPPLEMENTARY DATA

- SUPPLEMENTARY DATA
