# Supplementary material for: Tight regulation of plant immune responses by combining promoter and suicide exon elements
Source: Nucleic Acids Res. 2015 Jul 2;43(14):7152–61. doi: 10.1093/nar/gkv655 (PMC4538838; doi:10.1093/nar/gkv655)
Supplement: SUPPLEMENTARY DATA [file supp_gkv655_nar-01317-x-2014-File008_revised.pdf]

# SUPPORTING INFORMATION

## Tight regulation of plant immune responses by combining promoter and suicide exon elements

Tania L. Gonzalez, Yan Liang, Bao N. Nguyen, Brian J. Staskawicz, Dominique Loqué, and Ming C. Hammond

**Table of Contents**

**SUPPLEMENTAL FIGURES.**

**FIGURE S1.** Schematics of gene regulation with dexamethasone (Dex) induction.

**FIGURE S2.** Gene constructs used for transient transformations of *Nicotiana* plants in this study.

**FIGURE S3.** The E/P insertion site for *avrBs2-HyP5SM-HA* does not splice efficiently.

**FIGURE S4.** *N. benthamiana* transgenic for Bs2 shows HR development from *6xUAS::avrBs2-HyP5SM-HA* over time, without *Os*L5.

**FIGURE S5.** RT-PCR showing SP-I from *avrBs2-HyP5SM-HA* visible in the absence of *Os*L5.

**FIGURE S6.** Resistance gene and effector gene controls for the hypersensitive response.

**FIGURE S7.** Western blot time course of AvrBs2-HA and *Os*L5-6xHis protein expression from dual and single regulated constructs.

**FIGURE S8.** RT-PCR analysis of *ATR1Δ51-HyP5SM-FLAG* constructs and HR time course.

**FIGURE S9.** Transient expression of the multi-gene plasmid pTKan C59 shows hypersensitive response and protein.

**FIGURE S10.** Induced HR in transgenic *A. thaliana* plants expressing RPP1 and Dex-inducible ATR1Δ51 protein.

**FIGURE S11.** Transgenic *A. thaliana* plants show a Bs2/AvrBs2-dependent phenotype.

**TABLE S1.** Primers used for cloning and analysis.

**SUPPLEMENTAL METHODS.**

**I.** Cloning the HyP5SM cassette into a gene of interest.
  i. **FIGURE SM1**. Inserting the HyP5SM cassette into *avrBs2-HA*.

**II.** Cloning the multi-gene plasmids for faster generation of transgenic *Arabidopsis thaliana* plants.
  i. **TABLE S2**. Pieces used to generate the multi-gene pTKan vectors.
  ii. **FIGURE SM2**. The multi-gene pTKan constructs for transgenic *Arabidopsis*.

## Supplemental Figures

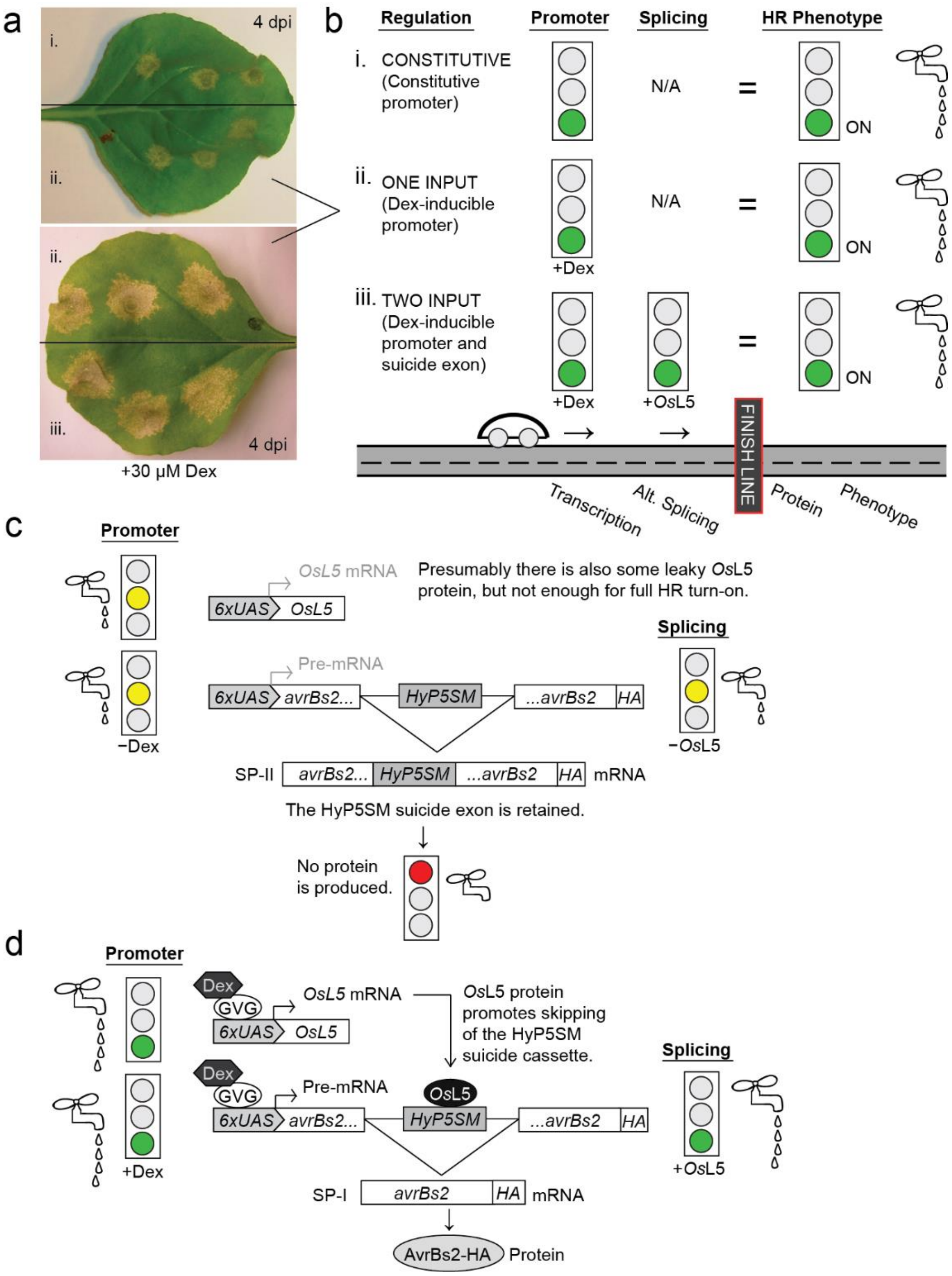


**FIGURE S1. Schematics of gene regulation with dexamethasone (Dex) induction.** (a) Schematic to complement Figure 1. Spot infiltrations of *Nicotiana benthamiana* leaves to compare i. pMD1 *avrBs2-HA*, ii. pTA7001 *avrBs2-HA*, and iii. pTA7001 *avrBs2-HyP5SM-HA*. All spots were co-infiltrated with p1776 *Bs2-HA*. The bottom leaf was also co-infiltrated with pTA7001 *OsL5-6xHis* on both sides. Leaves were sprayed with Dex. (b) Model of induced expression to complement Figure 1. (c), (d) Models showing how the two input regulation system (c) prevents leaky AvrBs2-HA protein and (d) can inducible promote AvrBs2-HA expression.

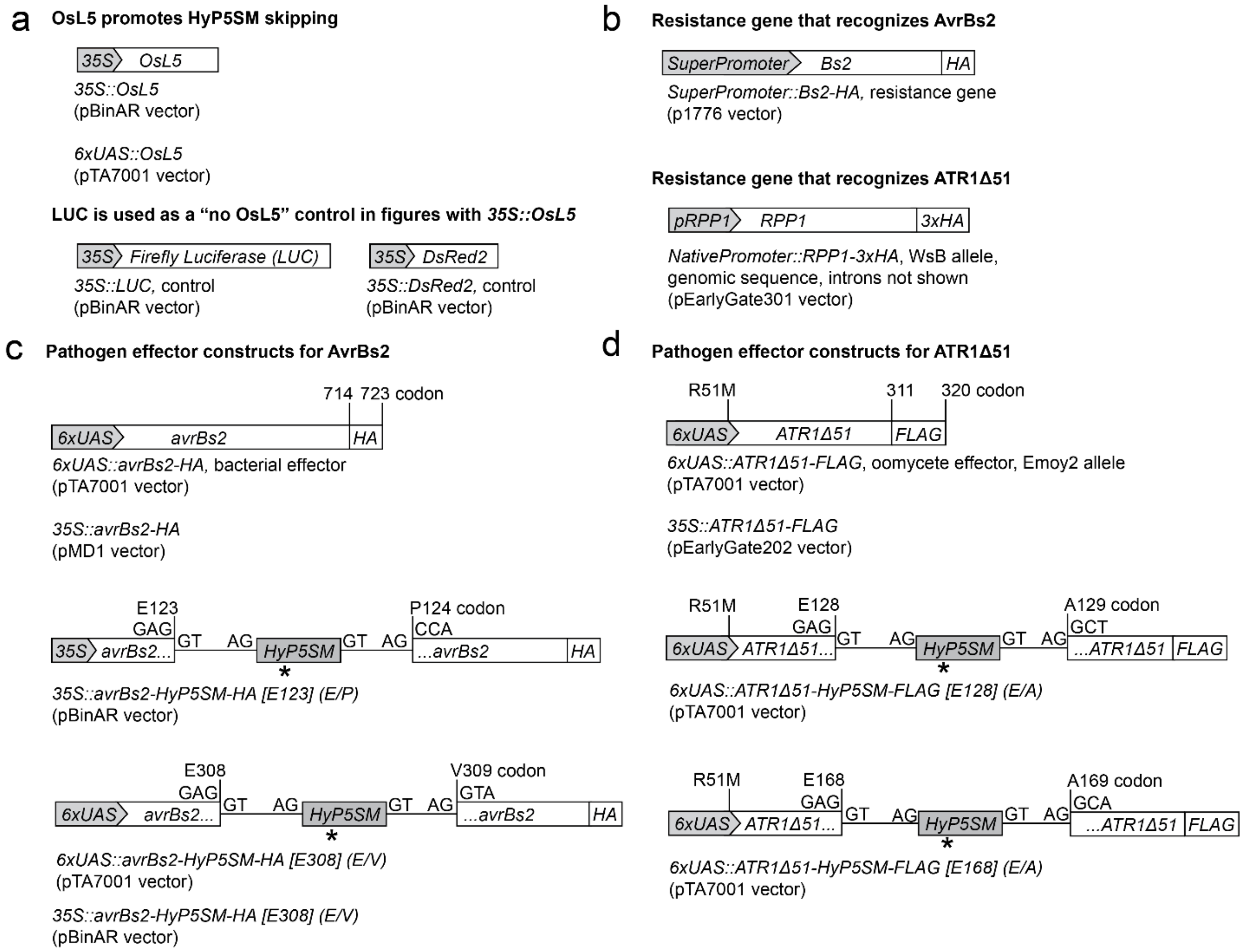


**FIGURE S2. Gene constructs used for transient transformations of *Nicotiana* plants in this study.** (a) Splicing effector and control constructs. (b) Resistance gene constructs. (c) Constructs for the bacterial effector AvrBs2 experiments. (d) Constructs for the eukaryotic pathogen effector ATR1Δ51 experiments. The N-terminal 51 amino acid truncation removes an eukaryotic exit signal.(1) The numbers above the constructs refer to amino acid codon positions. The dexamethasone inducible pTA7001 vector also includes *35S::GVG,* a transcription factor that binds the *6xUAS* promoter.(2) The p1776 vector has a strong constitutive chimeric octopine and manopine synthase promoter later renamed the "SuperPromoter" (3, 4).

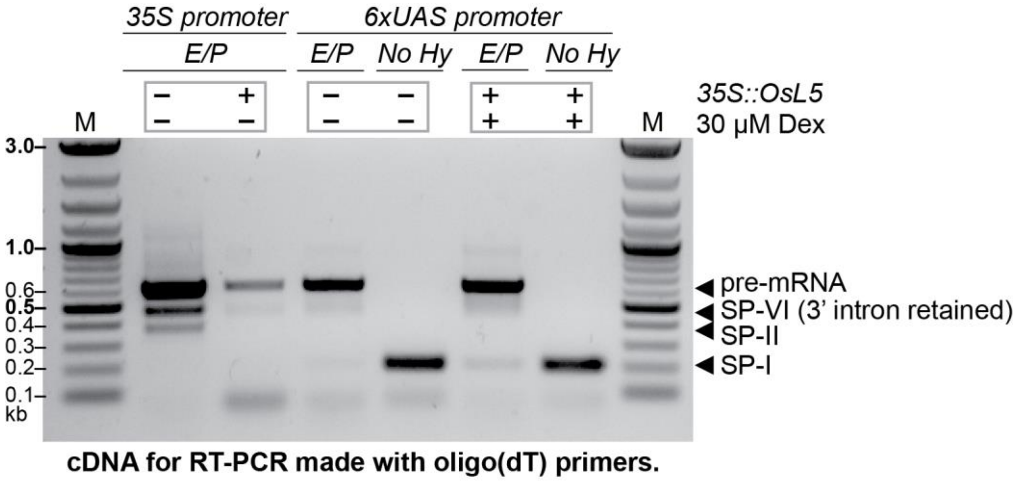


**FIGURE S3. The E/P insertion site for *avrBs2-HyP5SM-HA* does not splice efficiently.** RT-PCR for *avrBs2-HyP5SM-HA* (E/P insertion site) and *avrBs2-HA* (“No Hy”) samples from *N. benthamiana*. Co-expression of *35S::OsL5* was expected to produce the SP-I splice product (HyP5SM skipped), but *avrBs2-HyP5SM-HA* (E/P) exhibits poor HyP5SM exon skipping. Major intron-retained splice products were sequenced and identified as pre-mRNA and SP-VI (SP-II + 3’ intron). Gray boxes indicate that samples come from different halves of the same leaf. M = 2-log DNA ladder.

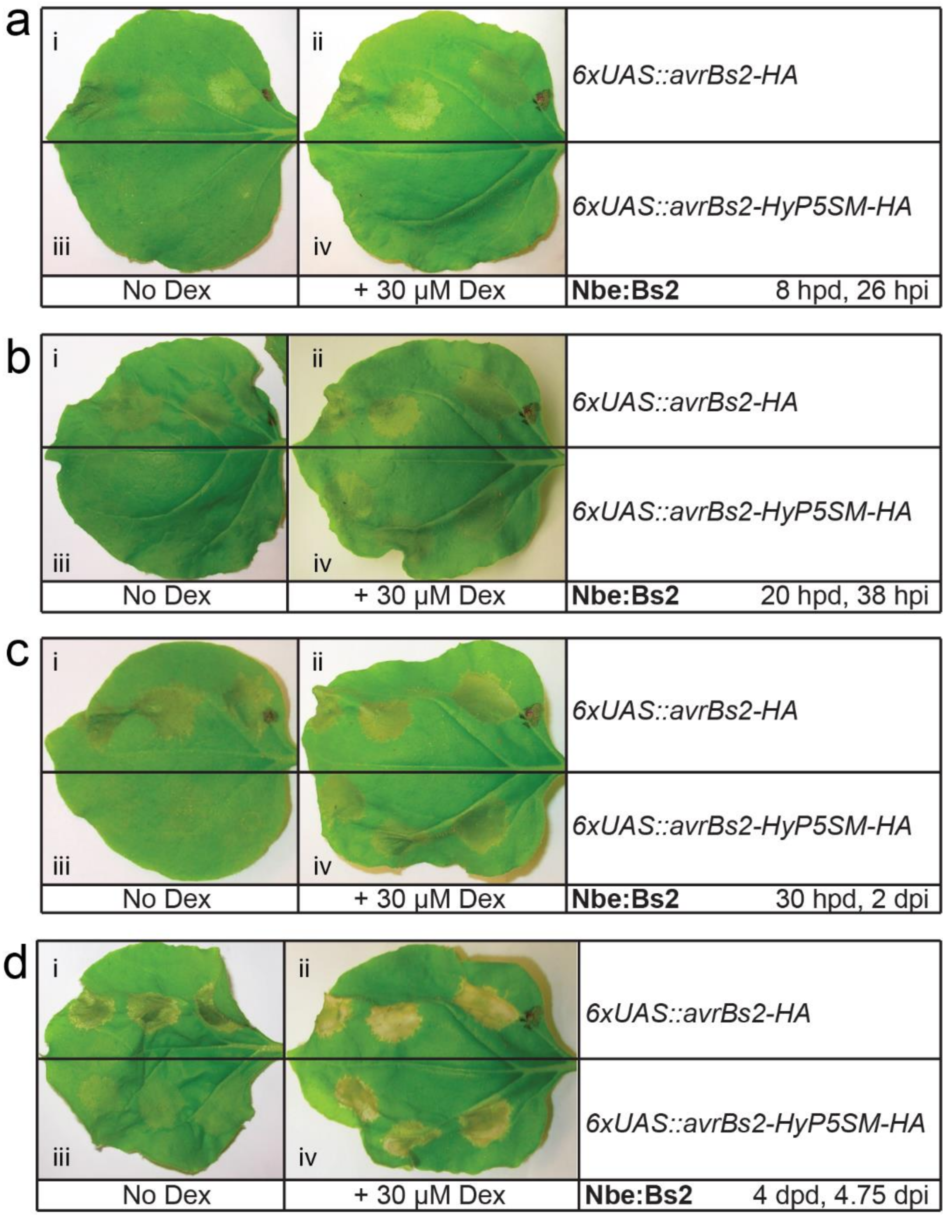


**FIGURE S4. *N. benthamiana* transgenic for Bs2 shows HR development from *6xUAS::avrBs2-HyP5SM-HA* over time, without *Os*L5.** Leaves of *N. benthamiana* stably expressing Bs2 were transiently infiltrated with either pTA7001 *avrBs2-HA* or pTA7001 *avrBs2-HyP5SM-HA*. They were either Dex induced or not induced at 18 hpi. Photos were taken (a) 8 h, (b) 20 h, (c) 30 h, and (d) 4 d later. With HyP5SM regulation, Dex-induced HR appears delayed 12 hours (a, b).

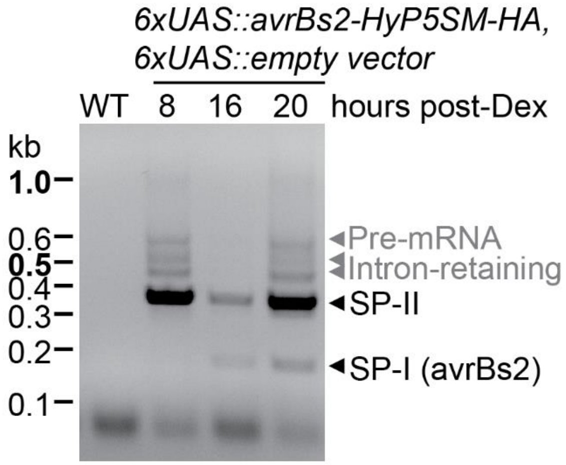


**FIGURE S5. RT-PCR showing SP-I from *avrBs2-HyP5SM-HA* visible in the absence of *Os*L5.** RT-PCR from *N. benthamiana* samples co-infiltrated with pTA7001 *avrBs2-HyP5SM-HA* and pTA7001 empty vector, in a parallel experiment accompanying the RT-PCR in Figure 4b. Samples were collected, processed, and run on an agarose gel alongside samples for Figure 4b. This image is cropped from the full gel.

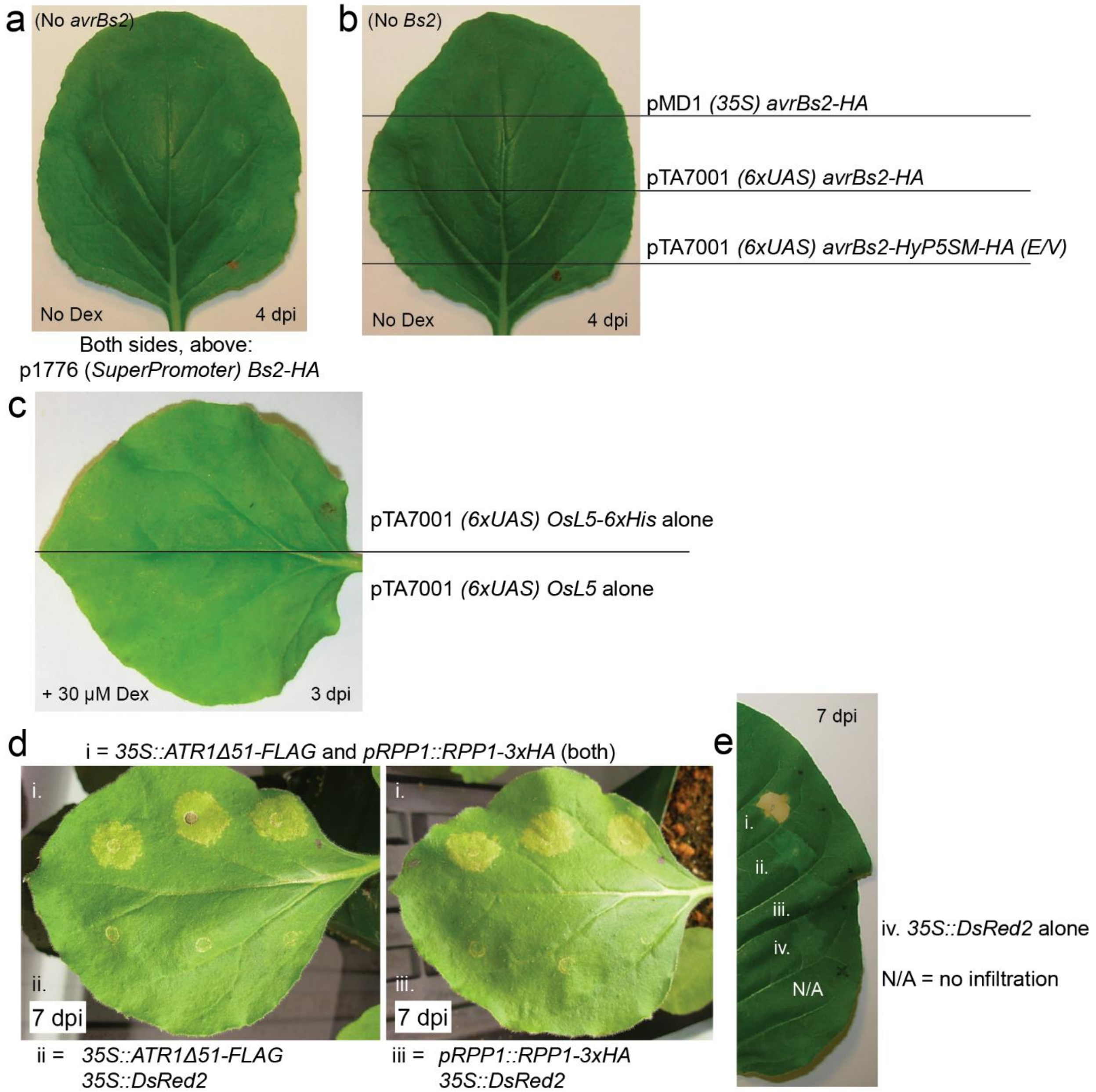


**FIGURE S6. Resistance gene and effector gene controls for the hypersensitive response.** *N. benthamiana* leaves were spot infiltrated or spot co-infiltrated with the indicated constructs using a needle-less syringe, with three spots on each leaf half. Total *Agrobacterium* (a-c) $OD_{600}$ = 0.75 or (d-e) $OD_{600}$ = 0.9. (a) *Bs2-HA* alone sometimes produces visible minor chlorosis (pale green color), but not the hypersensitive response. (b) *avrBs2* constructs do not initiate a hypersensitive response in the absence of the resistance gene *Bs2*. (c) The Dex-induced pTA7001 *OsL5* or *OsL5-6xHis* vectors alone do not result in a hypersensitive response. The red dot seen on all leaves near the stem was used to mark infiltrated leaves during the experiment. (d, e) Control infiltrations for ATR1 and RPP1. DsRed2 is added to keep $OD_{600}$ consistent. RPP1/ATR1Δ51-dependent HR is much stronger in (e) *N. tabacum* than (d) *N. benthamiana*.

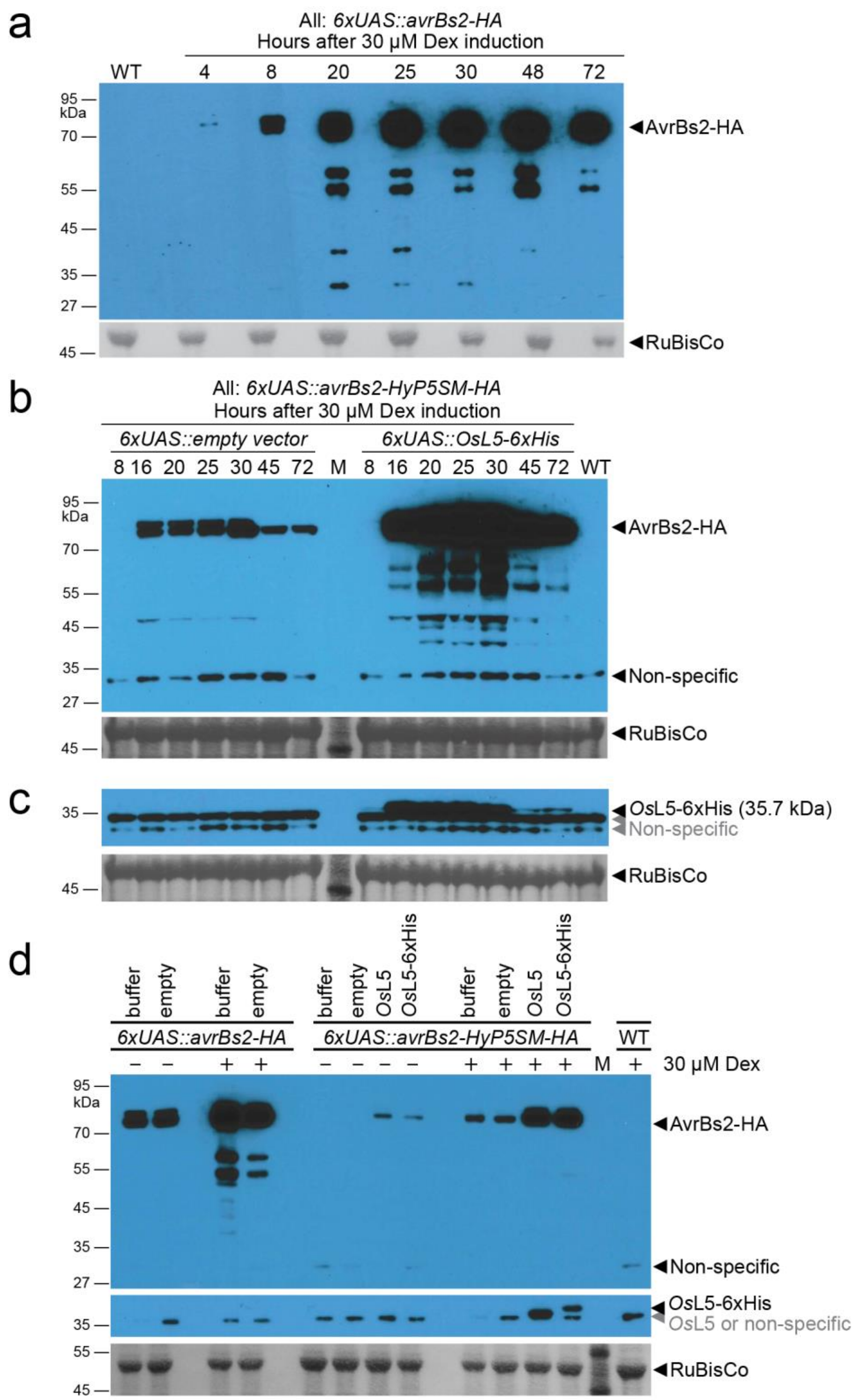


**FIGURE S7. Western blot time course of AvrBs2-HA and *Os*L5-6xHis protein expression from dual and single regulated constructs.** Western blot from *N. benthamiana* tissue transiently transformed, then induced with 30 µM Dex at 17 hpi. Tissue was collected at (a, b, c) the indicated times or (d) 24 h after treatment. (a, d) 5

µL or (b, c) 15 µL of crude proteins were run on 4-12% NuPAGE Bis-Tris gels with 1X MOPS buffer, then transferred to nitrocellulose. (a, b) Anti-HA blot shows AvrBs2-HA protein development from induced (a) pTA7001 *avrBs2-HA* alone or (b) pTA7001 *avrBs2-HyP5SM-HA* co-infiltrated either with pTA7001 empty vector or pTA7001 *OsL5-6xHis*. (c) Anti-*Os*L5-6xHis blot shows development of *Os*L5-6xHis protein (35.7 kDa) at slightly higher molecular weight than a non-specific band which may potentially be endogenous *Nb*L5 protein (estimated 34.6 kDa). (d) Anti-HA western blot (top) and anti-OsL5-6xHis western blot (middle) comparing the extent of protein expression from one-input and two-input regulation systems. *N. benthamiana* leaves were co-infiltrated in 1:1 mixes with the indicated pTA7001 *avrBs2* constructs, plus either buffer, pTA7001 empty vector, pTA7001 *OsL5*, or pTA7001 *OsL5-6xHis*. The "buffer" control displays the leakiness of pTA7001 without the confounding variable of an additional copy of the GVG transcription factor in pTA7001, while also maintaining equal final $OD_{600}$ of all *avrBs2* constructs. Lanes 3, 6, 11 are empty. Ponceau S stain of RuBisCo large subunit is shown as a loading control below each western blot. M = NEB #P7711 marker. WT = wild-type *N. benthamiana*.

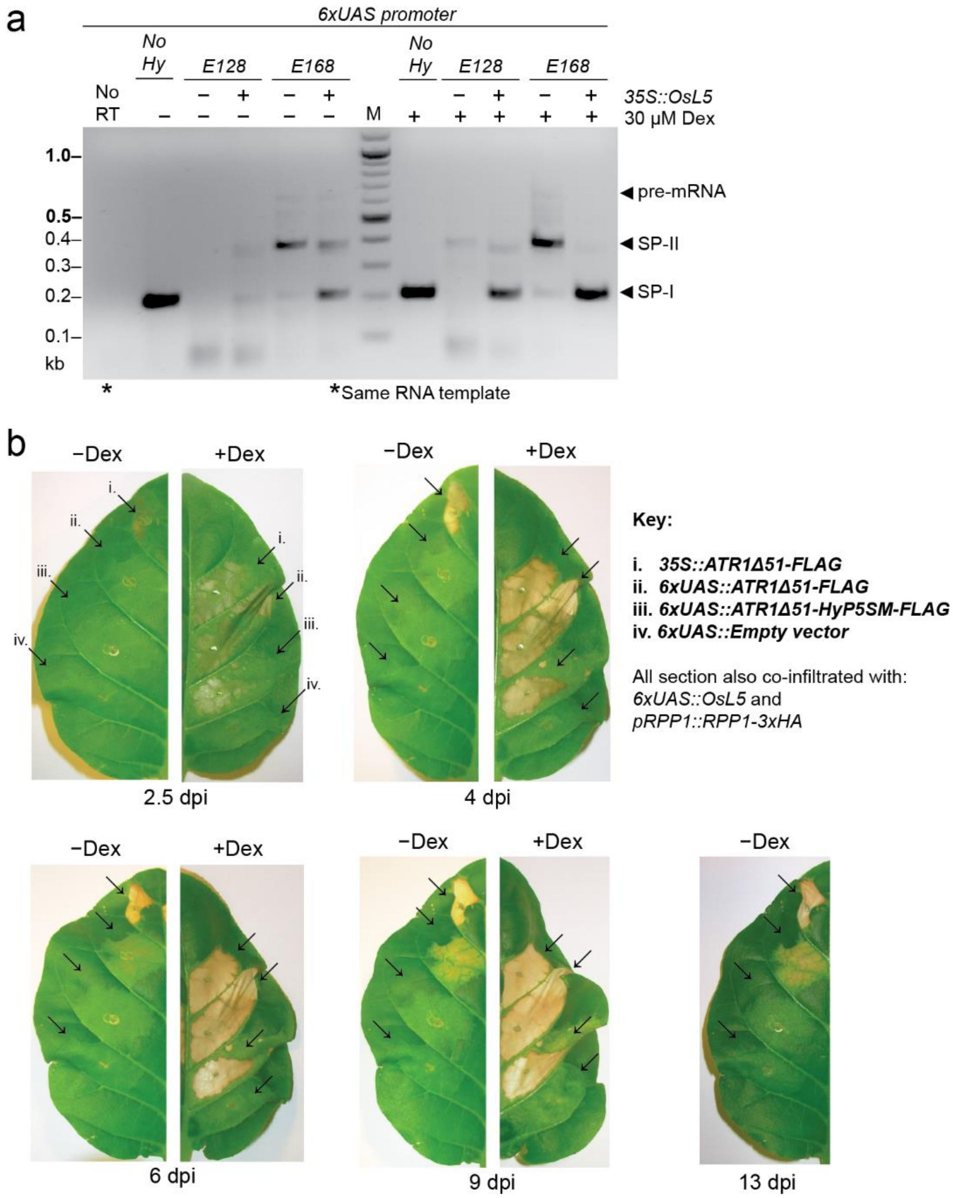


**FIGURE S8. RT-PCR analysis of *ATR1Δ51-HyP5SM-FLAG* constructs and HR time course.** (a) RT-PCR on 1.5% agarose gel of *6xUAS::ATR1Δ51-HyP5SM-FLAG* (with HyP5SM inserted after E128 or E168 codons) and *6xUAS::ATR1Δ51-FLAG* ("No Hy"). "No RT" is no reverse transcriptase control for genomic DNA contamination. RNA was extracted from *N. benthamiana* tissue from the same experiment described in Figure 5a. (b) Time course showing development of the hypersensitive response phenotype in transiently transformed *N. tabacum* leaves. The images follow two leaf halves through time. Because Dex is cell permeable, the two leaf halves are from different leaves. The leaky hypersensitive response from *6xUAS::ATR1Δ51-FLAG* is delayed, but apparent around 6 dpi.

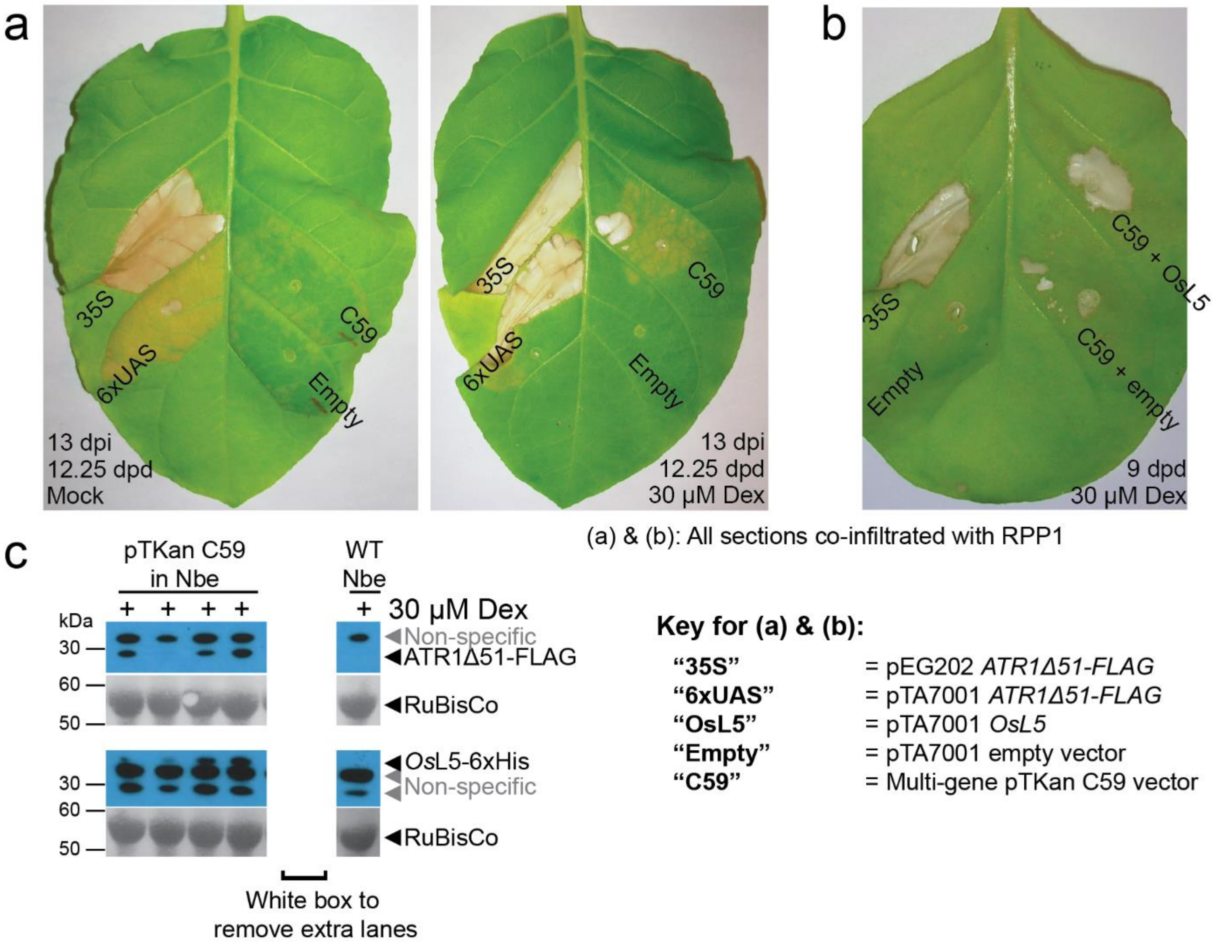


**FIGURE S9. Transient expression of the multi-gene plasmid pTKan C59 shows hypersensitive response and protein.** (a) Infiltrations to test "C59", the multi-gene construct pTKan C59 (which lacks RPP1, but contains Dex-inducible *ATR1Δ51-HyP5SM-FLAG* and *OsL5-6xHis*). All sections were co-infiltrated with pEG301 *RPP1-3xHA* in a 1:1 mix. The mock-induced leaf shows no hypersensitive response from pTKan C59. With Dex, the hypersensitive response is induced. (b) To investigate why the total hypersensitive response seems to be weaker from pTKan C59, *N. tabacum* was co-infiltrated with 1:1:1 mixes of RPP1, the indicated ATR1 or empty construct, and either pTA7001 *OsL5* or additional pTA7001 empty vector (which, although empty after the Dex-inducible promoter, still contains *35S::GVG* transcription factor). (c) *N. benthamiana* was transiently transformed with pTKan C59 and Dex-induced to promote protein expression. Western blots show expression of ATR1Δ51-FLAG (top: anti-FLAG) and *Os*L5-6xHis protein (bottom: anti-*Os*L5-6xHis) from four biological replicates. Below each western blot is a Ponceau S stain of RuBisCo.

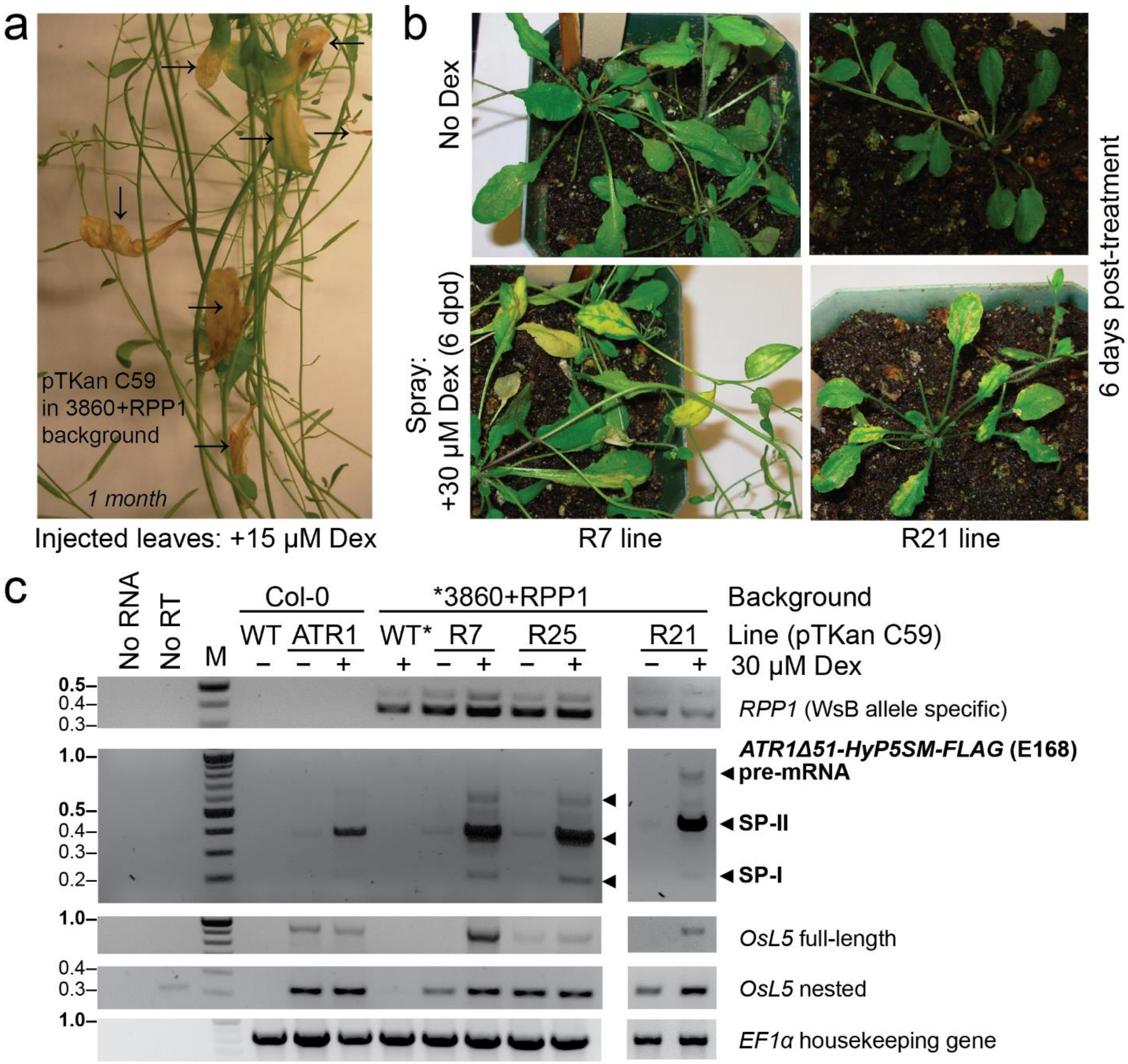


**FIGURE S10. Induced HR in transgenic *A. thaliana* plants expressing RPP1 and Dex-inducible ATR1Δ51 protein.** Plants are as described in Figure 6. (a) Injecting individual leaves of a bolting and early flowering plant with 15 µM Dex (see arrows) and growing in humid conditions on long days resulted in accelerated senescence of the induced leaves relative to the rest of the plant, but slower cell death relative to transient transformations (weeks instead of days). (b) Spraying late rosette-stage plants with 30 µM Dex and moving the plants to open growth carts on constant light resulted in faster cell death. These additional images of lines R7 (multiple rosettes in one pot) and R21 (single rosettes) accompany Figure 6b. (c) RT-PCR analysis of different plant lines. Col-0 WT contains no transgenes, Col-0 ATR1 contains pTKan C59 but no RPP1, WT* is 3860+RPP1 background line for R7, R25, and R21, which contain pTKan C59. The higher band observed for *RPP1* is an intron-retaining spliced product. Pre-mRNA, SP-I, and SP-II are observed for *ATR-HyP5SM-FLAG*. A nested PCR was performed on the PCR product from full-length *OsL5*, resulting in stronger signal.

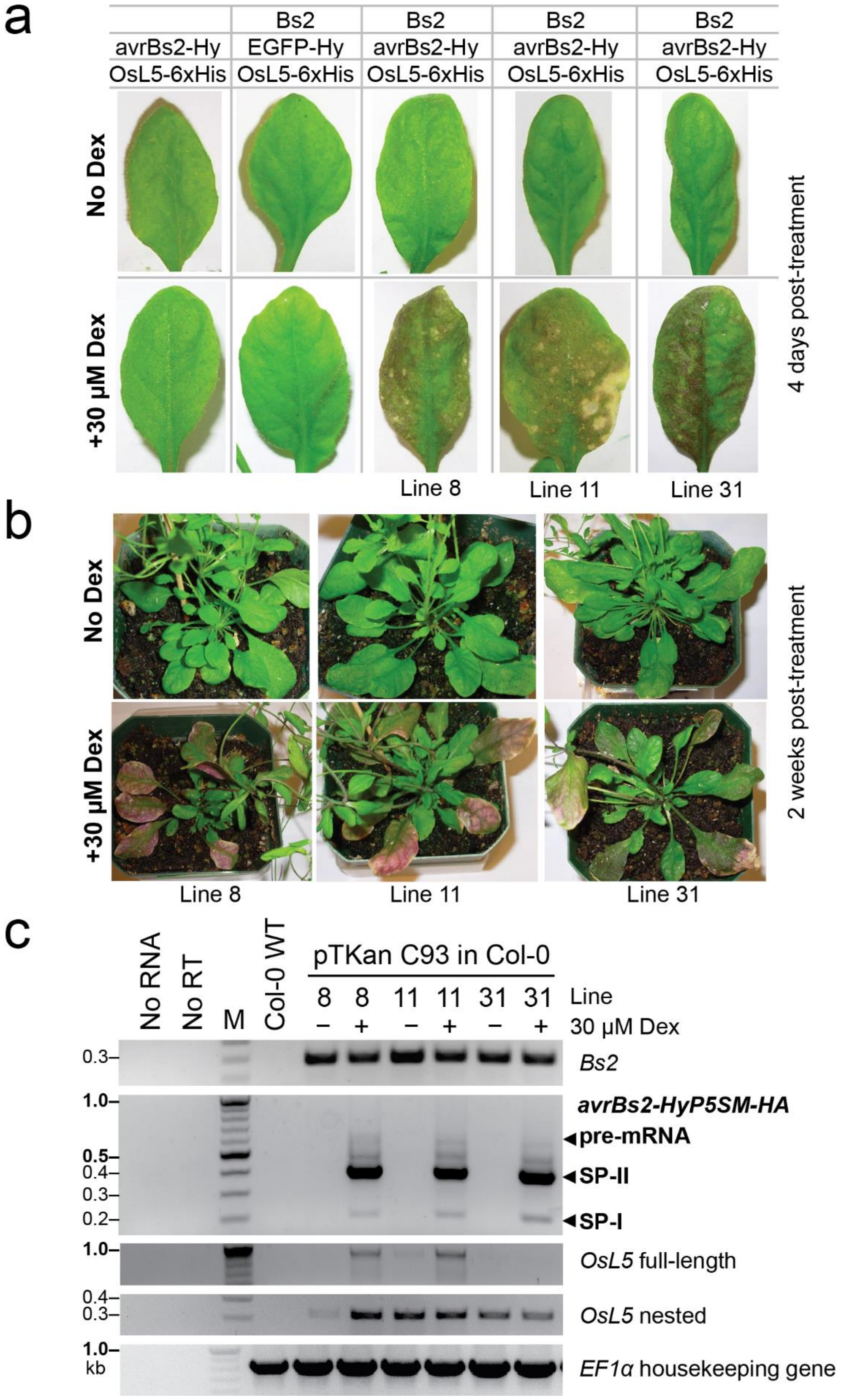
a
avrBs2-Hy
OsL5-6xHis
Bs2
EGFP-Hy
OsL5-6xHis
Bs2
avrBs2-Hy
OsL5-6xHis
Bs2
avrBs2-Hy
OsL5-6xHis
Bs2
avrBs2-Hy
OsL5-6xHis
No Dex
+30 µM Dex
4 days post-treatment
Line 8
Line 11
Line 31
b
No Dex
+30 µM Dex
2 weeks post-treatment
Line 8
Line 11
Line 31
c
No RNA
No RT
M
Col-0 WT
pTKan C93 in Col-0
8 8 11 11 31 31 Line
– + – + – + 30 µM Dex
0.3–
Bs2
1.0–
avrBs2-HyP5SM-HA
pre-mRNA
0.5–
0.4–
0.3–
0.2–
SP-II
SP-I
1.0–
OsL5 full-length
0.4–
0.3–
OsL5 nested
1.0–
kb
EF1α housekeeping gene

**FIGURE S11. Transgenic *A. thaliana* plants show a Bs2/AvrBs2-dependent phenotype.** (a) Transgenic plants expressing some combination of *6xUAS::avrBs2-HyP5SM-HA* or *6xUAS::EGFP-HyP5SM* (negative control), *6xUAS::OsL5-6xHis,* and *p35S::Bs2-3xFLAG*. Three second generation independent lines are shown for the plants containing both the *avrBs2* and the *Bs2* genes (made with pTKan C93 in Col-0). Dex induction stimulates Bs2/AvrBs2-dependent leaf darkening to a purple color. (b) Rosette images showing the progression of the phenotype, 2 weeks after treatment. (c) RT-PCR analysis of different plant lines. Col-0 WT contains no transgenes and is the background line for R8, R11, and R31, which contain pTKan C93 (Bs2, AvrBs2-HyP5SM-HA, OsL5). RNA was extracted from leaf tissue collected from the same plants before and 1 day after 30 μM Dex spray. Pre-mRNA, SP-I, and SP-II are observed for *AvrBs2-HyP5SM-HA*. A nested PCR was performed on the PCR product from full-length *OsL5*, resulting in stronger signal.

**TABLE S1.** Primers used for cloning and analysis.

Sequence Legend:

**lowercase bold** = restriction enzyme (RE) site

lowercase & not bold = random nucleotides for efficient RE digest or overlapping sequence for infusion cloning

lowercase with gray highlight = att site for BP recombination reaction

UPPERCASE = primer sequence

<u>UPPERCASE UNDERLINE</u> = point mutation

***UPPERCASE BOLD ITALIC*** = overlap with HyP5SM

F = forward primer; R = reverse primer

| **NAME** | **EXPERIMENT; 5'→3' SEQUENCE** | | | **SITE** |
|---|---|---|---|---|
| *>>> RT-PCR analysis of splice products. Primers are to amplify all splice products unless otherwise specified.* | | | | |
| | | ***avrBs2-HyP5SM-HA* (E/V)** | | |
| TLG36 | | CGGAAAACTCGCTGGCGTCCA | F | |
| TLG58 | | TGCGAATCACCAACGGCATTTCAC | R | |
| | | ***avrBs2-HyP5SM-HA* (E/V)**, SP-I specific | | |
| TLG83 | | ACGTCGAGGTAAGTTCCGATGG | F | |
| TLG84 | | AGCATCTGCTCCACACCG | R | |
| | | ***avrBs2-HyP5SM-HA* (E/P)** | | |
| TLG26 | | AGACCCTGCAAGGCAAG | F | |
| TLG57 | | TGCGACCTTGTTGTGCATCGATCA | R | |
| | | ***ATR1Δ51-HyP5SM-FLAG* (E128)** | | |
| GL35 | | GAACGGGATGATTTGATTGGCGAG | F | |
| TLG81 | | ACTGCTTCCTCCAATCGGTGC | R | |
| | | ***ATR1Δ51-HyP5SM-FLAG* (E168)** | | |
| TLG82 | | CACTAGGCTAGTAACAACCTATTCGG | F | |
| GL36 | | GGGTGCGAAAAAGTCAACATCGTG | R | |
| *>>> Other RT-PCR primers for A. thaliana samples* | | | | |
| | | ***RPP1* (WsB allele specific, amplifies coding sequence and intron-retaining product)** | | |
| TLG155 | | GCTCTACATGAGAGACTGCAAGG | F | |
| TLG156 | | GCGTTTCCAAAAGAGGGAAGC | R | |
| | | ***Bs2* resistance gene** | | |
| TLG191 | | GATTGTCGGGATGGGAGGCA | F | |
| TLG192 | | ACGCCATCCCACACTTCACA | R | |
| | | ***EF1alpha* housekeeping gene from *A. thaliana*** | | |
| MS62 | | GCTCTATGGAAGTTCGAGACC | F | |
| MS63 | | GTGTGGCAATCGAGAACTGG | R | |
| | | ***OsL5* full-length** | | |
| TLG47 | | atta**ctcgag**ATGGGAGGGTTTGTCAAGACCC | F | XhoI |
| TLG52 | | tatgct**actagt**TCACTCATCATCCTCTTCCTCGTC | R | SpeI |
| | | ***OsL5* internal product, for nested PCR** | | |

| | | | | |
|---|---|---|---|---|
| TLG86 | | CTCACGCTCCGTGGTTTGGACCAGG | F | |
| TLG87 | | CTCAGGTTCCTCCTCCGCCATAGACC | R | |
| *>>> Other RT-PCR primers for N. benthamiana samples* | | | | |
| | | ***OsL5* internal product optimized for specificity** | | |
| TLG88 | | ACCGTGTCTTTGGTGCCCTCAAG | F | |
| TLG87 | | CTCAGGTTCCTCCTCCGCCATAGACC | R | |
| *>>> Extension primers to insert HyP5SM cassette into avrBs2-HA at E/V codon site (GAA/GTC → GAG/GTA). E=E308.* | | | | |
| TLG43 | | ***ATGGTTTTCACTCTTTTGGTGTGTAG***GTAAGTTCCGATGG CGTGCCGGTGTT | F | |
| TLG44 | | ***CTCATGACAAGAGGATGCATAAATCTAC***CTCGAC GTCCAGCTCCAGATTGCGGTA | R | |
| *>>> Extension primers to insert HyP5SM cassette into avrBs2-HA at E/P codon site (GAG/CCG→GAG/CCA). E=E123.* | | | | |
| TLG32 | | ***CACTCTTTTGGTGTGTAG***CCAGTGTATCTGGATACCGCC | F | |
| TLG33 | | ***GACAAGAGGATGCATAAATCTAC***CTCCAGTGTGCCGGCA GCAA | R | |
| TLG34 | | GCTGCCGGCACACTGGAG***GTAGATTTATGCATCCTCTTGT C*** | F | |
| TLG35 | | CGGTATCCAGATACACTGG***CTACACACCAAAAGAGTGAA AACC*** | R | |
| *>>> Extension primers to insert HyP5SM cassette into ATR1Δ51-FLAG at E128 codon site (E/A; GAA/GCT→GAG/GCT)* | | | | |
| GL31 | | ***CACTCTTTTGGTGTGTAG***GCTCTCGCCACTAGGCTAG | F | |
| GL30 | | ***GACAAGAGGATGCATAAATCTAC***CTCATCATAGGTATCAT GGAGTGG | R | |
| *>>> Extension primers to insert HyP5SM cassette into ATR1Δ51-FLAG at E168 codon site (E/A; GAA/GCA→GAG/GCA)* | | | | |
| GL33 | | ***CACTCTTTTGGTGTGTAG***GCAGTGGCATCACTATGGAA | F | |
| GL32B | | ***GACAAGAGGATGCATAAATCTAC***CTCCTCCAATCGGTGC G | R | |
| *>>> Primers to amplify the full-length HyP5SM cassette, including introns, for use in 3-piece-ligation PCR, previously published* (5) | | | | |
| DNA37 | | GTAGATTTATGCATCCTCTTGTCATGAG | F | |
| DNA38 | | CTACACACCAAAAGAGTGAAAACCAT | R | |
| *>>> Cloning into pTA7001 (into the XhoI/SpeI site), XbaI ends are compatible with SpeI ends* | | | | |
| | | ***avrBs2-HA; avrBs2-HyP5SM-HA* (E/P and E/V)** | | |
| TLG45 | | tta**ctcgag**ATGCGTATCGGTCCTCTGCAACCTTC | F | XhoI |
| TLG46 | | catt**tctaga**CTACGCATAGTCAGGAACATCGTATGGGTAATCC | R | XbaI |
| | | ***ATR1Δ51-FLAG; ATR1Δ51-HyP5SM-FLAG* (E128 and E168)** | | |
| GL28 | | tta**ctcgag**ATGGCGCAGACAGCTC | F | XhoI |
| GL29 | | catt**tctaga**TTAGCCTTTGTCGTCATCG | R | XbaI |
| | | ***OsL5*** | | |
| TLG47 | | atta**ctcgag**ATGGGAGGGTTTGTCAAGACCC | F | XhoI |
| TLG52 | | tatgct**actagt**TCACTCATCATCCTCTTCCTCGTC | R | SpeI |
| | | ***OsL5-6xHis*** | | |
| TLG47 | | atta**ctcgag**ATGGGAGGGTTTGTCAAGACCC | F | XhoI |
| TLG80 | | cagtg**actagt**TCAGTGGTGATGATGGTGATGA | R | SpeI |
| *>>> Cloning into pBinAR (into the KpnI/SalI site)* | | | | |
| | | ***avrBs2-HyP5SM-HA* (E/P and E/V)** | | |
| TLG53 | | tta**ggtacc**ATGCGTATCGGTCCTCTGCAACCTTC | F | KpnI |
| TLG54 | | catt**gtcgac**CTACGCATAGTCAGGAACATCGTATGGGTA | R | SalI |
| | | **Primers to make *OsL5-6xHis* from *OsL5* template** | | |
| TLG51 | | atta**ggtacc**ATGGGAGGGTTTGTCAAGACCC | F | KpnI |

| | | | | |
|---|---|---|---|---|
| TLG65 | | ctt**gtcgac**TCAGTGGTGATGATGGTGATGACCGGATCCCTCA TCATCCTCTTCCTCGTC | R | SalI |
| *>>> Sequencing primers* | | | | |
| | | **pTA7001** | | |
| TLG39 | | AACGACGGCCAGTGAATTCTCGAAG | F | |
| | | **pBinAR** | | |
| BinFor | | CTATCCTTCGCAAGACCCTTCCTCTATA | F | |
| BinRev | | AATATCATGCGATCATAGGCGTCT | R | |
| *>>> Primers to generate multi-gene pTKan plasmids* | | | | |
| | | **CaMV 35S promoter** | | |
| YL1 | | CATGGAGTCAAAGATTCAAAT | F | |
| YL2 | | CCCG**cctagg**AGTCCCCCGTGTTCTCTCCAA | R | AvrII |
| | | **pNOS-DsRed2-tNOS** | | |
| YL3 | | ggggatcctctagagGATACATGAGAATTAAGGGAG | F | |
| YL4 | | tccgcggacgtccc**gggccc**GAGCTTGCATGCCGGTCG | R | ApaI |
| | | **GVG-tRbcsE9** | | |
| YL5 | | acgggggactcctag**ctcgagcctagg**ATGAAGCTACTGTCTTCTAT CG | F | XhoI AvrII |
| YL6 | | gagggctgtcacccgAGTGTTTTACTCCTCATATTAACTTCGG | R | |
| | | **p6xUAS promoter** | | |
| YL7 | | gaggagtaaaacactCGGGTGACAGCCCTC | F | |
| YL8 | | acaggctagc**cctagg**CAGCGTGTCCTCTCCAAATG | R | AvrII |
| | | ***OsL5-6xHis*** | | |
| YL9 | | acaagtttgtacaaaaaagcaggcttcATGGGAGGGTTTGTCAAGAC | F | attB1 |
| YL10 | | acaactttgtatagaaaagttgggtgTCAGTGGTGATGATGGTGATG | R | attB4 |
| | | ***ATR1Δ51-HyP5SM-FLAG (E168)*** | | |
| YL11 | | acaactttgtataataaagttggcATGGCGCAGACAGCTC | F | attB3 |
| YL12 | | accactttgtacaagaaagctgggtcTTAGCCTTTGTCGTCATCGTC | R | attB2 |
| | | ***AmpR* for insertion alone** | | |
| YL13 | | cccgggtaccaagctAATTCTTGAAGACGAAAGGG | F | |
| YL14 | | tagactcgag**aagctt**TACCAATGCTTAATCAGTGA | R | HindIII |
| | | ***p35S-Bs2-3xFlag-tNOS* for insertion with *AmpR*** | | |
| YL15 | | cccgggtaccaagctCATGGAGTCAAAGATTCAAATAG | F | |
| YL16 | | ccctttcgtcttcaagaattGATCTAGTAACATAGATGACACC | R | |
| | | ***AmpR* for insertion with *p35S-Bs2-3xFlag-tNOS*** | | |
| YL17 | | ctatgttactagatcAATTCTTGAAGACGAAAGGG | F | |
| YL14 | | tagactcgag**aagctt**TACCAATGCTTAATCAGTGA | R | HindIII |
| | | ***avrBs2-HyP5SM-HA*** | | |
| YL18 | | acaactttgtataataaagttggcATGCGTATCGGTCCTCTGC | F | attB3 |
| YL19 | | accactttgtacaagaaagctgggtaCTACGCATAGTCAGGAACATCG | R | attB2 |
| | | ***eGFP-HyP5SM*** | | |
| YL20 | | acaactttgtataataaagttggcATGTCTAGAGTGAGCAAGGGC | F | attB3 |
| YL21 | | accactttgtacaagaaagctgggtaCTTGT ACAGCT CGTCC ATGCC | R | attB2 |

## SUPPLEMENTAL METHODS

### I. Cloning the HyP5SM cassette into a gene of interest

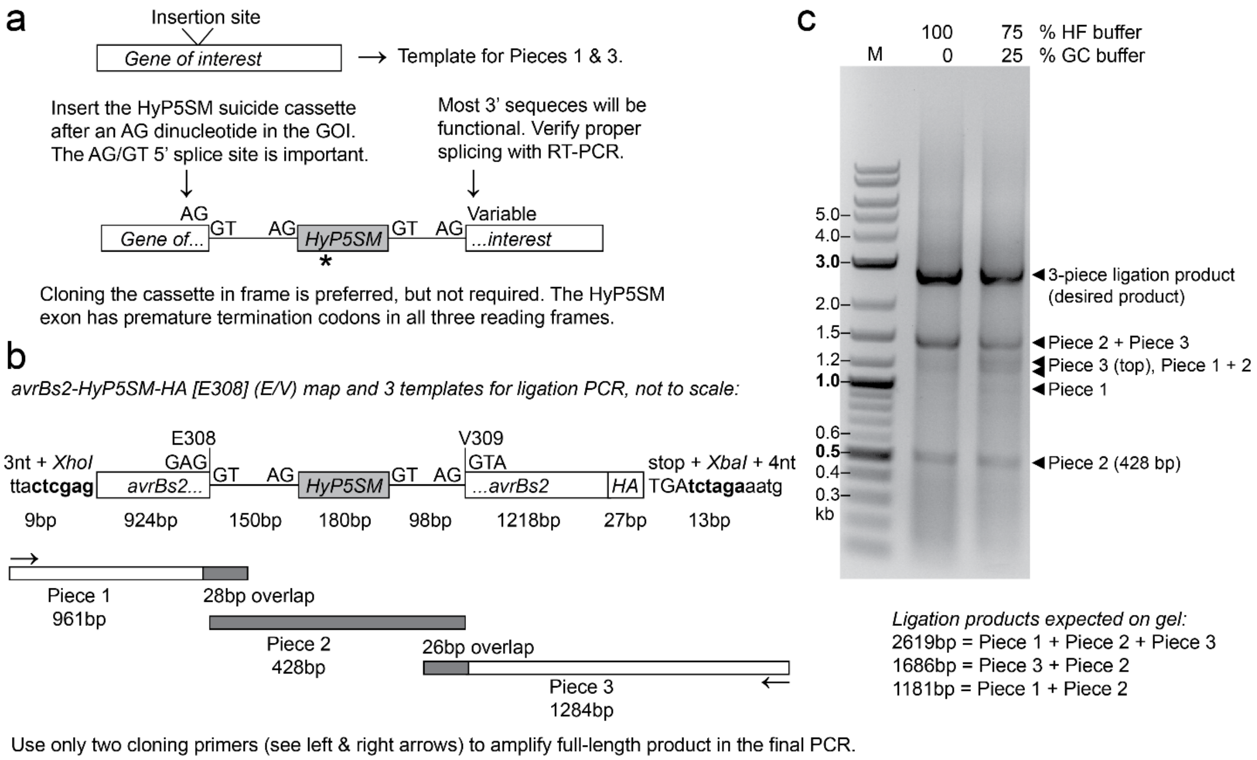


**FIGURE SM1. Insertion of the HyP5SM cassette into *avrBs2-HA*.** (a) Chose an insertion site after an AG dinucleotide (5). The 5' intron provides the GT dinucleotide to complete the minimal 5' splice site. (b) HyP5SM is introduced into the gene of interest using 3-piece-ligation PCR. The three template pieces and final product are shown. (c) 1% agarose gel showing the 3-piece-ligation products. "HF buffer" and "GC buffer" refer to the buffers for Phusion HF DNA Polymerase (New England Biolabs).

*Step 1: Choose an insertion site in the sequence of the gene of interest*.

The most conserved splice site is the 5' splice site.(6) The HyP5SM cassette must be cloned after an **AG** dinucleotide in the gene of interest.(5) The following silent mutations may be used if cloning the cassette in frame. Fortunately, dicot plants do not have strong codon preferences for these amino acids.(7)

| | |
|---|---|
| Glutamate (E): | GAA → GAG |
| Glutamine (Q): | CAA → CAG |
| Lysine (K): | AAA → AAG |

The 3' splice site is variable. The 3' splice site closest to the consensus sequence is **GU**, but many other sites will work as well. The endogenous 3' splice site for the *A.*

*thaliana* P5SM cassette is **AGA**.(8) See Hickey *et al.* 2012 Figure 2 for mutational analysis of the 3' splice site for HyP5SM in *EGFP*.(5)

For best results, clone 2-3 versions of the gene of interest with HyP5SM in different insertion sites and check the splicing of each construct with RT-PCR. Gel extract and sequence the splice products to verify that the HyP5SM cassette is splicing as expected. Choose the best insertion site empirically.

Example with *avrBs2:*

***E/V (GAA/GTG → GAG/GTA)*** – Silent mutations introduce the essential AG dinucleotide for the 5' splice site and make the 3' splice site closer to the HyP5SM endogenous 3' splice site (AGA). This insertion site is also close to the minimal consensus 3' splice site for plants (GTN).(6)

*Step 2: Make the three templates for 3-piece-ligation PCR (also called extension PCR).*

Make three separate dsDNA templates with a high fidelity polymerase.

Piece 1:

[NNN][XhoI site][ATG---*avrBs2 first part*---GAG][GT--5'intron overhang--

Template: *avrBs2-HA*

Primers: The *avrBs2* forward cloning primer (TLG45). The reverse primer (TLG44) anneals to *avrBs2* at the chosen insertion site and adds an overhang. This same sequence overhang can be used for cloning HyP5SM into other genes.

Piece 2:

[GT---5'intron---AG][HyP5SM exon][GT---3'intron---AG]

Template: Any gene with the full HyP5SM cassette already cloned into it. In this case, we used *EGFP-HyP5SM* (5).

Primers: Primers DNA37 (forward) and DNA38 (reverse).

Piece 3:

---3'intron overhang---AG][GTA---*avrBs2 second part*---][HA][XbaI][NNNN]

Template: *avrBs2-HA*

Primers: The forward primer (TLG43) anneals to *avrBs2* at the insertion site and adds an overhang. The *avrBs2* reverse primer (TLG46) adds an XbaI site.

Gel extract each product from an agarose gel. Calculate the molar concentration of the PCR products.

*Step 3: Perform 3-piece-ligation PCR with a high fidelity polymerase.*

Templates: The three PCR products in a 1:1:1 molar ratio. For *avrBs2-HyP5SM-HA (E/V)* cloning, the final concentration of each template was approximately 0.15 nM.

Primers: The *avrBs2-HA* forward and reverse cloning primers (TLG45, TLG46). The overhangs in the templates also act as primers.

*Step 4:* The desired full-length product was gel purified, inserted into a cloning vector (Invitrogen pCR2.1 TOPO), and sequenced to confirm that the junctions were correct and that there were no frame shifts. The sequence perfect product was then cloned into a binary vector for plant expression.

## II. Cloning the multi-gene plasmids for faster generation of transgenic *Arabidopsis thaliana* plants

*Step 1: Construction of the pTKan expression vector*.

The p35S promoter was amplified by PCR from pRT100 (9) and cloned into pCRblunt (Invitrogen) to generate *pBlunt-p35S*. *pTKan-p35S-GW* binary vector was generated by digesting *p35S* promoter from *pBlunt-p35S* with XhoI/AvrII, and then inserted between XhoI/AvrII restriction sites of *pTKan-GW-R1R2* vector (10). The *pNOS-DsRed2-tNOS* sequence was synthesized and fused into ApaI site of *pTKan-GW-R1R2* vector by in-fusion cloning (In-Fusion® HD, Clontech) to generate *pTKan-pNOS-DsRed2-tNOS-p35S-GW-R1R2* which was subsequently digested with AvrII restriction enzyme to fuse with *GVG-tRbcsE9* and *p6xUAS* fragments. The in-fusion product is the expression vector *pTKan-pNOS-DsRed2-tNOS-p35S-GVG-tRbcsE9-p6xUAS-GW-R1R2*.

*Step 2: Construction of the entry vectors*

To generate Gateway entry clones, attB or attBr-flanked PCR products were cloned into pDONR 221 vectors with corresponding attP or attPr sites (MultiSite Gateway®Pro, Invitrogen) by BP recombination reaction. In this way, *OsL5-6xHis* was

cloned into pDONR221 P1-P4 vector; *eGFP-HyP5SM, ATR1Δ51-HyP5SM-FLAG (E168 insertion site)*, and *avrBs2-HyP5SM-HA* were cloned into pDONR221 P3-P2 vector, respectively; *tG7-p6xUAS* was cloned into pDONR221 P4r-P3r vector. pDORN221-*tG7-AmpR-pUAS* R4-R3 and pDONR221-*tG7- p35S-Bs2-3xFlag-tNOS-AmpR-pUAS* R4-R3 constructs were generated by in-fusion cloning (In-Fusion® HD, Clontech). Briefly, AmpR alone or *AmpR* together with *p35S-Bs2-3xFlag-tNOS* was inserted inbetween *tG7* and *p6xUAS* at HindIII site of the pDONR221 *tG7-p6xUAS* R4-R3 vector in single in-fusion cloning reactions. The PCR template of *p35S-Bs2-3xFlag-tNOS* was provided by pMD1 Bs2-3xFLAG from the Staskawicz lab. The insertion of AmpR helps to stabilize the existence of two *p6xUAS* promoters in the final expression constructs.

*Step 3: Construction of the multi-gene plasmids*

In a MultiSite Gateway reaction (MultiSite Gateway®Pro, Invitrogen), genes or fragments of interest in pDONR221 P1-P4 vector, pDONR221 P4r-P3r vector and pDONR221 P3-P2 vector were connected and incorporated into GW R1-R2 expression vector in a sequential manner. MultiSite Gateway reaction was carried out with the expression vector *pTKan-pNOS-DsRed2-tNOS-p35S-GVG-tRbcsE9-p6xUAS-GW-R1R2* and various entry vectors from Step 1 and Step 2. Entry vector combinations for the final expression constructs were listed in the following table.  LR Clonase™ II Plus enzyme mix and equal molar of each component construct were used in the MultiSite Gateway reaction according to the manufacturer's manual.

**TABLE S2.** Pieces used to generate the multi-gene pTKan vectors.

| **Final construct** | **DNA element 1** | **DNA element 2** | **DNA element 3** |
|---|---|---|---|
| **C59** | pDONR221-OsL5-6xHis L1L4 | pDONR221-tG7-AmpR-pUAS R4R3 | pDONR221-ATR1Δ51-HyP5SM-FLAG L3L2 |
| **C92** | pDONR221-OsL5-6xHis L1L4 | pDONR221-tG7-p35S-Bs2-3xFlag-tNOS-AmpR-pUAS R4R3 | pDONR221-avrBs2-HA L3L2 |
| **C93** | pDONR221-OsL5-6xHis L1L4 | pDONR221-tG7-p35S-Bs2-3xFlag-tNOS-AmpR-pUAS R4R3 | pDONR221-avrBs2-HyP5SM-HA L3L2 |
| **C226** | pDONR221-OsL5-6xHis L1L4 | pDONR221-tG7-AmpR-pUAS R4R3 | pDONR221-avrBs2-HyP5SM-HA L3L2 |
| **C228** | pDONR221-OsL5-6xHis L1L4 | pDONR221-tG7-p35S-Bs2-3xFlag-tNOS-AmpR-pUAS R4R3 | pDONR221-eGFP-HyP5SM  L3L2 |

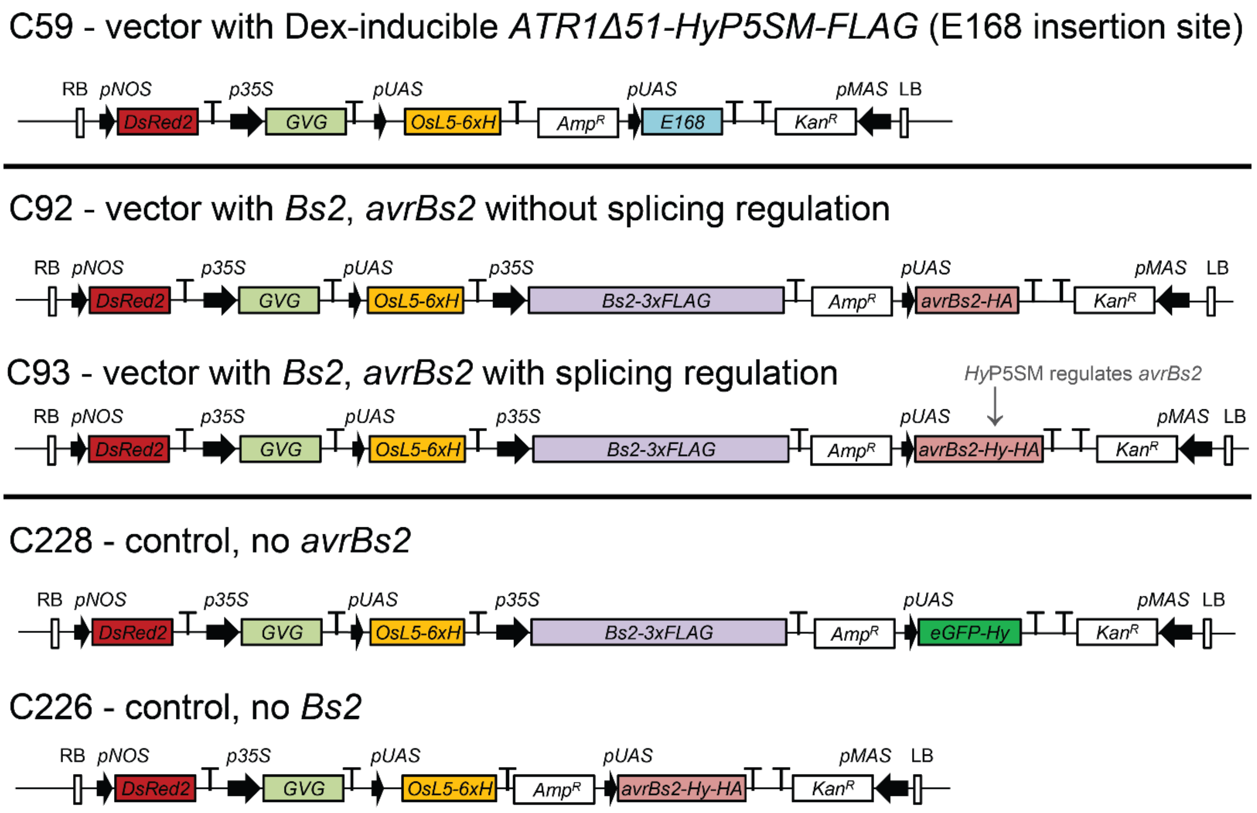


**FIGURE SM2. The multi-gene pTKan constructs used for transgenic *Arabidopsis*.** See supplemental methods for cloning description. Abbreviations include pNOS = nopaline synthase promoter, p35S = Cauliflower Mosaic Virus 35S promoter, pUAS = p6xUAS promoter (induced by GVG transcription factor in presence of dexamethasone), 6xH = 6xHis tag, E168 = *ATR1Δ51-HyP5SM-FLAG* (with the splicing cassette inserted after codon E168), pMAS = mannopine synthase promoter, Hy = HyP5SM cassette.

## SUPPLEMENTAL REFERENCES

1. Krasileva, K. V, Dahlbeck, D. and Staskawicz, B.J. (2010) Activation of an Arabidopsis resistance protein is specified by the in planta association of its leucine-rich repeat domain with the cognate oomycete effector. *Plant Cell*, **22**, 2444–2458.

2. Aoyama, T. and Chua, N.-H. (1997) A glucocorticoid-mediated transcriptional induction system in transgenic plants. *Plant J.*, **11**, 605–612.

3. Day, B., Dahlbeck, D., Huang, J., Chisholm, S.T., Li, D. and Staskawicz, B.J. (2005) Molecular Basis for the RIN4 Negative Regulation of RPS2 Disease Resistance. *Plant Cell*, **17**, 1292–1305.

4. Lee, L.-Y., Kononov, M.E., Bassuner, B., Frame, B.R., Wang, K. and Gelvin, S.B. (2007) Novel plant transformation vectors containing the superpromoter. *Plant Physiol.*, **145**, 1294–300.

5. Hickey, S.F., Sridhar, M., Westermann, A.J., Qin, Q., Vijayendra, P., Liou, G. and Hammond, M.C. (2012) Transgene regulation in plants by alternative splicing of a suicide exon. *Nucleic Acids Res.*, **40**, 4701–4710.

6. Simpson, G.G. and Filipowicz, W. (1996) Splicing of precursors to mRNA in higher plants: mechanism, regulation and sub-nuclear organisation of the spliceosomal machinery. *Plant Mol. Biol.*, **32**, 1–41.

7. Murray, E.E., Lotzer, J. and Eberle, M. (1989) Codon usage in plant genes. *Nucleic Acids Res.*, **17**, 477–498.

8. Hammond, M.C., Wachter, A. and Breaker, R.R. (2009) A plant 5S ribosomal RNA mimic regulates alternative splicing of transcription factor IIIA pre-mRNAs. *Nat. Struct. Mol. Biol.*, **16**, 541–549.

9. Töpfer, R., Matzeit, V., Gronenborn, B., Schell, J. and Steinbiss, H.H. (1987) A set of plant expression vectors for transcriptional and translational fusions. *Nucleic Acids Res.*, **15**, 5890.

10. Eudes, A., George, A., Mukerjee, P., Kim, J.S., Pollet, B., Benke, P.I., Yang, F., Mitra, P., Sun, L., Cetinkol, O.P., et al. (2012) Biosynthesis and incorporation of side-chain-truncated lignin monomers to reduce lignin polymerization and enhance saccharification. *Plant Biotechnol. J.*, **10**, 609–620.
